# Discovery and Characterization of a Nonacidic Small-Molecule Inhibitor of the Sodium-Coupled Dicarboxylate Transporter NaCT

**DOI:** 10.1021/acs.jmedchem.5c02711

**Published:** 2026-04-28

**Authors:** Andrew Quigley, Jörg T. Kley, Alexander Pautsch, Stefan G. Kauschke, Amy Chu-Antypas, Annamaria Tessitore, Claire Strain-Damerell, Leela Shrestha, Dieter Wiedenmayer, Nicola A. Burgess-Brown, Oleg Fedorov, Elisabeth P. Carpenter

**Affiliations:** † Centre for Medicines Discovery, NDM Research Building, Roosevelt Drive, Headington, Oxford OX3 7FZ, U.K.; ‡ Membrane Protein Laboratory, Research Complex at Harwell, Diamond Light Source, Harwell Oxford, Didcot OX11 0DE, U.K.; § Global Medicinal Chemistry, Boehringer Ingelheim Pharma GmbH & Co. KG, 88397 Biberach, Germany; ∥ Cardio-Renal-Metabolic Disease Discovery Research, Boehringer Ingelheim Pharma GmbH & Co. KG, 88397 Biberach, Germany

## Abstract

Citrate is a key metabolic intermediate that regulates both glycolysis and lipid metabolism. While most citrate is produced in mitochondria, some is imported from the bloodstream by the SLC13 family of sodium-coupled transporters. Among these, hepatic citrate transporter NaCT (SLC13A5) mediates citrate uptake and is a potential therapeutic target for metabolic disorders by limiting hepatic citrate uptake. However, the loss of NaCT expression or function is linked to neonatal encephalopathy and cancer risk, underscoring the need for selective tools to study NaCT biology. Here, we report the development of a potent and selective piperidinecarboxamide-based chemical probe, BI01383298, along with an inactive analog, BI01372674, and an alternative probe, BI01455810. BI01383298 is highly potent (IC_50_ = 25 nM), shows exceptional selectivity (>1000-fold over other SLC13 transporters), and has robust cellular activity, whereas BI01372674 shows no measurable activity. Together, these compounds provide valuable tools for probing the physiological and pathological roles of NaCT in metabolism and disease.

## Graphical abstract

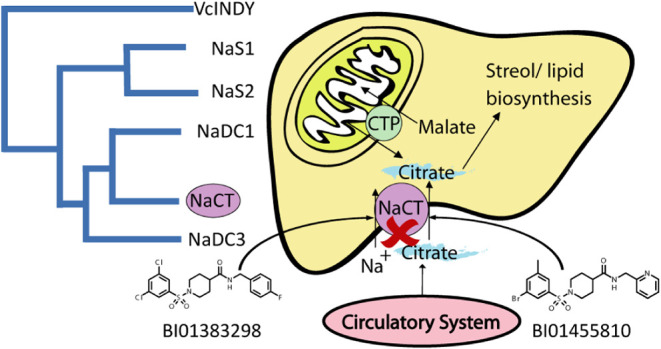

## Introduction

Citrate is a critical intermediate of the Krebs cycle, a precursor of cholesterol and lipid biosynthesis, an effector of glycolysis, and an important signaling molecule. Modulation of cytosolic citrate levels has a direct effect on both glucose metabolism and energy production.^1,2^ Citrate regulates glycolysis through the inhibition of phosphofructokinase and influences lipogenesis through the activation of acetyl-CoA carboxylase.^3,4^

Cytosolic citrate levels are controlled by the mitochondrial citrate transporter CTP and the high-affinity sodium-citrate cotransporter (NaCT).^5–8^ NaCT transports citrate from the circulatory system into hepatocytes and may be particularly important under nutrient-limited conditions and as a defense against metal ion toxicity.^9^ Mouse models have highlighted the potential of NaCT as a target for obesity and diabetes, with knockout mice protected from adiposity.^10^ Reduced lipid concentrations were observed in an siRNA study,^11^ and a substrate analog was used to show lower blood glucose levels in mice.^12^ Mutations to the homologous I’m Not Dead Yet (INDY) gene were found to be associated with an increased lifespan in *Drosophila melanogaster* ^13,14^ and *Caenorhabditis elegans*.^15^

NaCT, NaDC1, and NaDC3 belong to the SLC13 family, along with NaS1 and NaS2. NaS1 and NaS2 are sodium-sulfate transporters and are not thought to transport Krebs cycle intermediates, whereas NaCT, NaDC1, and NaDC3 cotransport Krebs cycle intermediates such as citrate, succinate, and α-ketoglutarate (reviewed in refs. 16–18 ). NaCT, NaDC1, and NaDC3 are expressed in distinct locations. NaCT is predominantly expressed in the plasma membrane of hepatocytes, with lower levels of expression also observed in the brain and testes.^5^

The transport activity of human and other primate NaCT has been shown to be stimulated by lithium, whereas rodent NaCTs are inhibited.^7,19^ This is relevant to the clinical usage of lithium as a treatment for bipolar disorder, as concentrations of lithium in patients are at physiologically relevant levels for the stimulation of NaCT activity. Additionally, lithium is usually formulated as lithium citrate, further increasing circulating citrate concentrations. Strikingly, it has been reported that patients receiving lithium treatment sometimes report significant weight gain, and an increase in circulating triglycerides can be observed.^20^ Lithium chloride has also been shown to lead to lipid accumulation in HepG2 and RAW264.7 cells, as well as the livers of adult and larval zebrafish.^21^

More recently, mutations in NaCT have been linked to early infantile epileptic encephalopathy, Kohlschütter–Tönz syndrome, teeth and bone development, and metabolic disease, while silencing of the SLC13A5 gene inhibits proliferation of human hepatocarcinoma cells.^22–27^ While evidence supporting NaCT inhibition suggests that pharmacological modulation of this transporter may offer therapeutic potential for treating obesity, metabolic disorders such as metabolic dysfunction-associated fatty liver disease (MAFLD), and certain cancers (see recent reviews: refs. 17,28–30 ), the presence of disease-associated mutations underscores the need to understand the biological consequences of NaCT-targeted therapeutics.

In 2015, Huard et al. were the first to report the discovery of a potent and selective NaCT inhibitor, PF-06649298 (1),^12^ contrasting with previously reported inhibitors of NaCT that featured limited potency and selectivity.^31,32^ The chemical scaffold of PF-06649298 (1) and related compounds are hydroxysuccinic acids (Figure 1), which resemble citrate, a substrate of NaCT. Not surprisingly, substrate-competitive binding to NaCT has been reported for PF-06649298 (1).^12,33^ Further NaCT inhibitors structurally related to PF-06649298 (1) were recently reported.^34^ Originally it was thought that the mechanism by which the hydroxysuccinic acid compounds inhibited NaCT was through competitive inhibition. However, it has emerged that the mechanism is more complex, with these compounds acting in an allosteric state-dependent manner with low affinities in the absence of citrate.^35^ Recently, the atomic resolution structure of NaCT was solved bound to PF-06649298 (1). This revealed the structural basis of NaCT inhibition with the inhibitor binding on the cytosolic side in the same position as the substrate, also supporting a competitive binding mechanism.^36^ In contrast to the hydroxysuccinic acid-based inhibitors such as PF-06649298, the structure of the potent NaCT inhibitor ETG-5773 (3) described by Zahn et al.^37^ represents a fundamentally different chemotype. ETG-5773 is structurally unrelated to citrate and is devoid of acidic functional groups and therefore does not mimic the endogenous substrate.^37^

**Figure 1:**
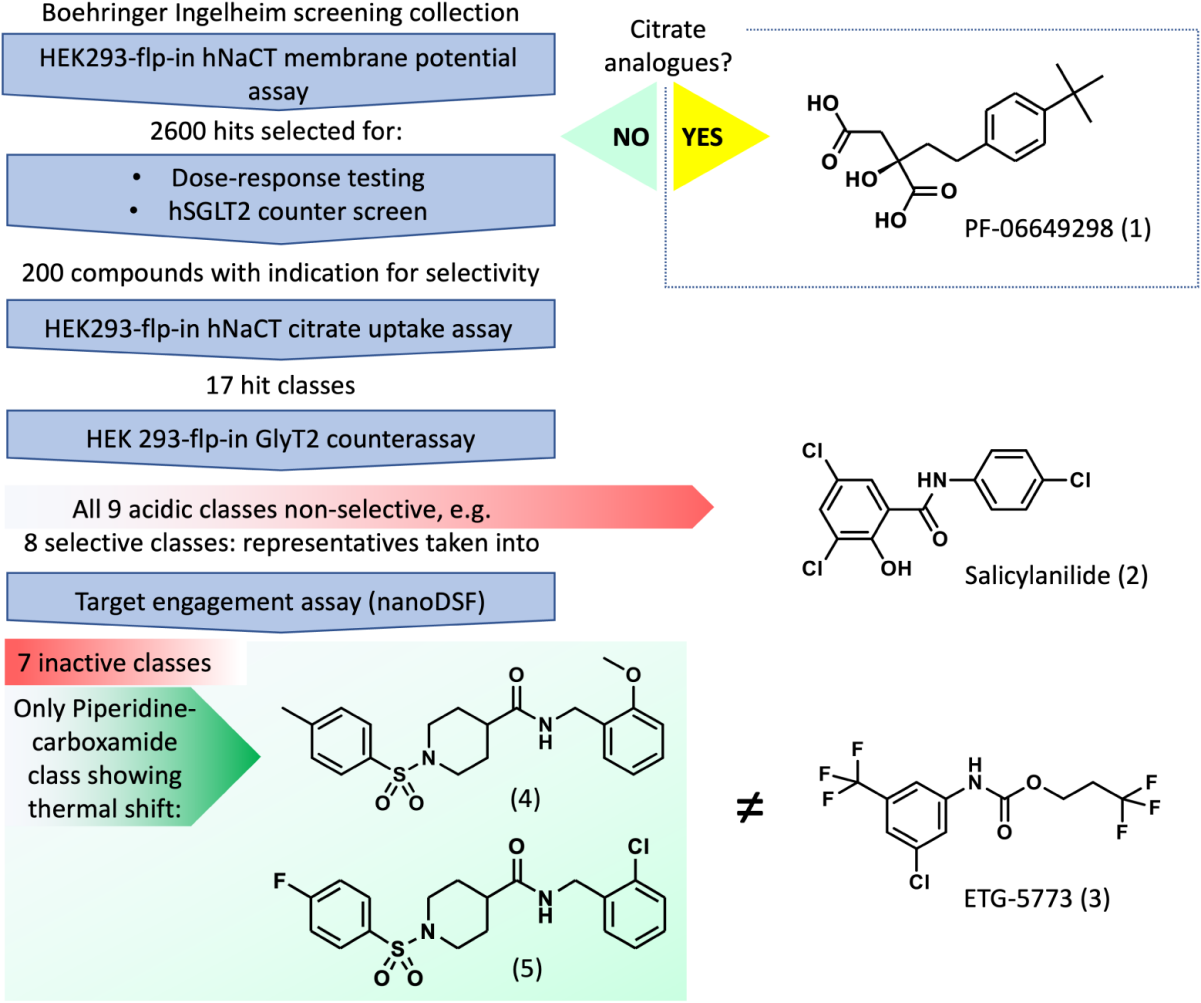
A schematic view of the screening cascade applied in the discovery of the piperidinecarboxamide class of NaCT inhibitors. The structure of the substrate analog PF-06649298 (1) is shown alongside devalidated hit classes represented by the salicylanilide (2) and the structurally unrelated NaCT inhibitor ETG-5773 (3). The selective piperidinecarboxamide class is represented by parts (4) and (5).

Here we report the discovery of a structurally unrelated chemotype of selective nonacidic NaCT inhibitors with a piperidinecarboxamide core. The highly potent series representative BI01383298 (6) shows high selectivity over both other dicarboxylate transporters of the SLC13 family and over 42 of a panel of 44 unrelated targets. Interestingly, we did not observe inhibitory activity against the murine homologue of NaCT. A structurally closely related negative control, BI01372674 (7), which was devoid of detectable potency against NaCT, is also reported. Beyond what has been disclosed in patent literature, in Boehringer Ingelheim’s open science portal opnMe^38^ and by others,^39^ we present basic structure–activity relationship data of the piperidinecarboxamide series and the characterization of BI01383298 (6), including evidence for direct target interaction and its impact on citrate metabolism. Furthermore, we confirm these characteristics by data generated with the more soluble analog BI01455810 (8).

## Results and Discussion

### Discovery of a Novel, Specific Human NaCT Inhibitor Chemotype

With the goal of identifying small molecule starting points for the generation of potent and selective inhibitors of human NaCT (hNaCT), around 900,000 compounds from Boehringer Ingelheim’s screening collection were screened using a membrane potential assay. Approximately 200 hits were identified with confirmed activity and indication for selectivity based on a human sodium-glucose cotransporter 2 (hSGLT2) counter screen. These compounds were subsequently screened in a citrate uptake assay using HEK293 cells overexpressing human NaCT (Figure 1).

After applying a potency cutoff of 10 μM in the citrate uptake assay, the remaining hits were distributed over 17 structural classes. Interestingly, all acidic hit classes (with calculated p*K*_a_ < 6) proved to be nonspecific, based on the human glycine transporter 2 (GlyT2) counter assay. GlyT2 was selected for the counter assay as a similar transporter but with different substrate specificity. We assumed that salicylanilides (exemplified by compound 2) and other acidic chemotypes had shown activity in the membrane potential assay due to mitochondrial uncoupling activity,^40,41^ rather than through interaction with human NaCT.

To complement the citrate uptake assays and assess whether potential inhibitors directly interact with hNaCT, we developed a thermostabilization assay that was validated using substrates of hNaCT. The assay uses purified, detergent-solubilized hNaCT, and thus observed thermal shifts are a result of a direct interaction with the protein-detergent complex. hNaCT stabilization was observed in the presence of sodium citrate and sodium succinate but not in the presence of sodium sulfate (Figure 2). This agrees with hNaCT acting as a dicarboxylate transporter with a preference for citrate over succinate.^6^ We also tested the effect of the presence of different cations such as lithium as this has previously been shown to activate transport.^7^ We found that in a 200 mM background of sodium, 50 mM lithium chloride significantly increased the thermostability of NaCT with a 2-fold increase in the thermal shift observed compared to sodium citrate and a 4-fold thermal shift compared to when 50 mM potassium citrate was added. A selection of representatives from 8 hit classes that met our criteria for potency (IC_50_ < 10 μM in the HEK293-Flp-in-hNaCT citrate uptake assay) and selectivity (inactive in the hGlyT2 counter assay) were tested at a concentration of 10 μM using the hNaCT thermostability assay previously described. Unexpectedly, we detected a thermal shift only within a single class, represented by structurally related piperidinecarboxamides 4 and 5 (Figure 1). Chemical structures are not disclosed for classes A–G, for which thermostabilization was not observed.

**Figure 2:**
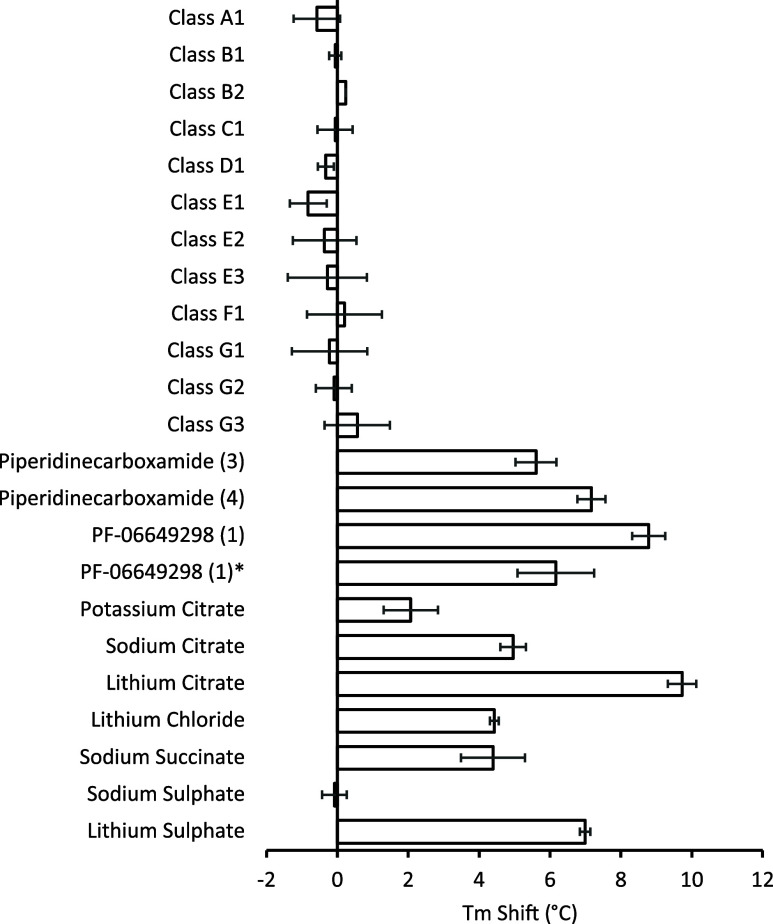
Thermostability of human NaCT based upon intrinsic tryptophan fluorescence as measured by nanodifferential scanning fluorimetry (DSF). Human NaCT thermostability in the presence of relevant substrate at 50 mM or compound class representatives at 10 μM (*n* = 9). Lithium citrate and sodium succinate were included as substrate-based controls (*n* = 9). PF-06649298 (1) was included as a previously identified inhibitor of human NaCT. *Racemic mixture of PF-06649298 (1) and its optical antipode (*n* = 3). Lithium sulfate (*n* = 4).

### Chemistry

The synthesis of piperidinecarboxamides (6–8) was achieved through standard amidation and deprotection steps, as outlined in Figure 3. The phosphinoxide-containing building block 22 was prepared by palladium-mediated C–P coupling of dimethylphosphinoxide with 4-iodobenzonitrile, followed by hydrogenation of the nitrile group. Further analogs (compounds 9–18) given in Table S1 were prepared analogously. ^1^H NMR spectra of compounds 6–8 are shown in the Supporting Information.

**Figure 3:**
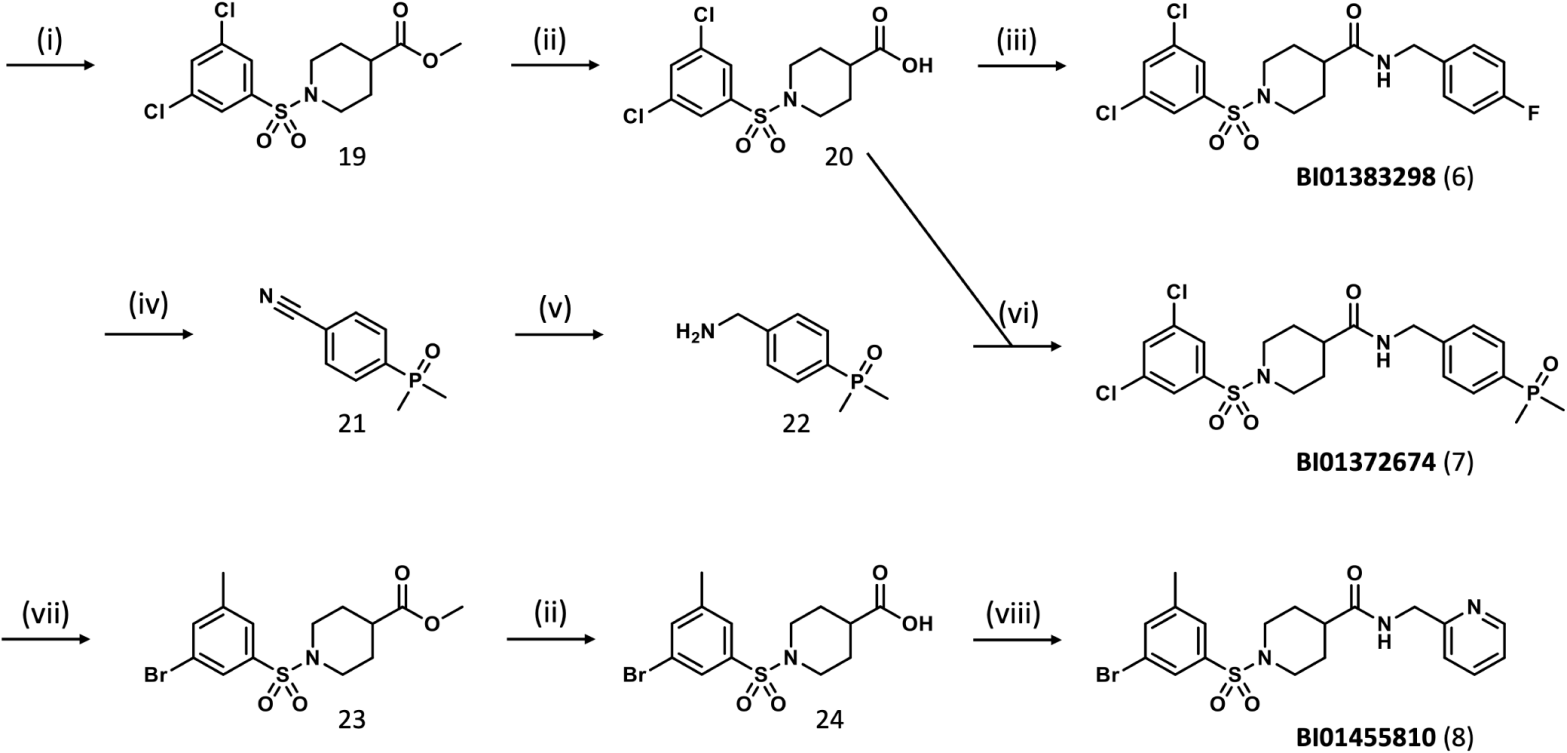
Synthesis and chemical structures of the tool compound BI01383298 (6), the inactive structural analog BI01372674 (7), and the more soluble but slightly less potent BI01455810 (8). Reaction conditions: (i) 3,5-dichlorobenzenesulfonyl chloride, piperidine-4-carboxylic acid methyl ester, NEt_3_, DCM, r.t.; (ii) NaOH, MeOH-H_2_O, 70 °C; (iii) 4-fluorobenzylamine, TBTU, NEt_3_, DMF, r.t.; (iv) 4-iodobenzonitrile, dimethylphosphinoxide, Pd_2_bda_3_, xantphos, CsCO_3_, MeCN; (v) H_2_, Raney-Ni, NH_3_-MeOH (vi) TBTU, NEt_3_, DMF, r.t.; (vii) 3-bromo-5-methylbenzenesulfonyl chloride, piperidine-4-carboxylic acid methyl ester, NEt_3_, DCM, r.t.; (viii) 2-(aminomethyl)­pyridine, TBTU, NEt_3_, DMF, r.t.

### Structure–Activity Relationship (SAR) of Human NaCT Inhibitors with Piperidinecarboxamide Core

An assay quantifying citrate uptake in human HepG2 cells was used to determine SAR. While even minor modifications of the central part of the piperidinecarboxamide led to a reduction in potency, we could rapidly improve the potency of our initial screening hits 4 and 5 by roughly 2 orders of magnitude through the introduction of small substituents in both the 3- and 5-positions of the phenylsulfonyl moiety (Figure 4; exemplified by compounds 9 and 10 in Table S1).

**Figure 4:**
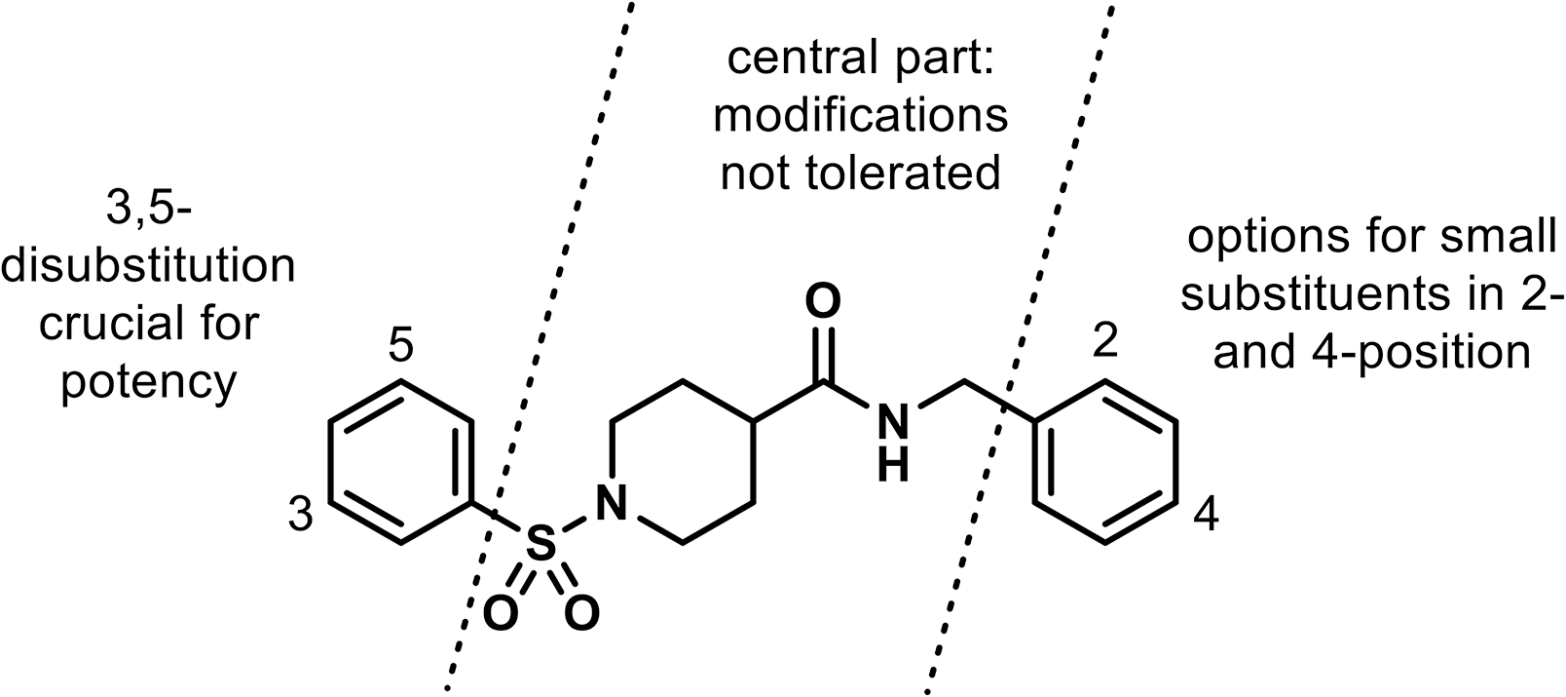
Schematic representation of structure–activity relationships for inhibition of human NaCT within the piperidinecarboxamide class. Potency data of representative compounds are given in the SI (tTable S1).

An extended amidation series varying the benzylamine moiety (right-hand side in Figure 4) revealed a rather steep SAR also in this region with some options for the addition of small substituents in the 2- and 4-positions of the benzyl unit with moderate influence on potency (exemplified by compounds 6, 9, 10, and 12 in Table S1). Within the structural type depicted in Figure 4, we identified several compounds, exemplified by BI01383298 (6), that fulfilled our criteria for potency as well as selectivity over the closely related NaDC1 and NaDC3.

With the less advantageous aqueous solubility of BI01383298 (6) (Figure 3, Table 1), we embarked on improving solubility while keeping potency. The introduction of polar or basic solubilizing substituents into either of the two phenyl moieties proved incompatible with high potency. This is illustrated by the completely inactive analog BI01372674 (7), which differs from BI01383298 (6) by only a single substituent exchange (dimethylphosphoryl vs fluorine). However, a complementary approach replacing the benzyl moiety by heteroarylmethyls turned out to be fruitful: within a series of pyridyl and thiazolyl analogs (exemplified by compounds 8, 14–18 in Table S1), we were pleased to identify decently soluble compounds with a less than 10-fold reduction in potency as observed for BI01455810 (8) (Figure 3, Table 1).

**Table 1:** Compound Characteristics for the Tool Compound BI01383298 (6), the More Soluble Analog BI01455810 (8), the Negative Control BI01372674 (7), and the Previously Described PF-06649298 (1)

	BI01383298 **(6)**	BI01455810 **(8)**	BI01372674 **(7)**	PF-06649298 **(1)**
Molecular weight [Da]	445.3	452.4	503.4	294.3
HEK293-hSLC13A5 IC_50_ [μM]	0.05 (±0.009)	0.285 (±0.016)	>100	13.3 (±13.0)
HepG2 IC_50_ [μM]	0.0245 (±0.004)	0.184 (±0.015)	>100	54 (±73.3)
HEK293-hSLC13A2 IC_50_ [μM]	>100	n.d.	n.d.	>100
HEK293-hSLC13A3 IC_50_ [μM]	>100	n.d.	n.d.	12
HEK293-mSLC13A5 IC_50_ [μM]	>100	>70	>70	8.9
HEK293-GLYT2 IC_50_ [μM]	>100	>100	n.d.	>100
Solubility @pH 2.2/4.5/6.8 [μg/mL]	<1/<1/<1	>100/83/67	77/75/70	>73/>74/>74
PAMPA permeability @pH 7 [10^–6^ cm/s]	3.8	5.1	0.15	0.011
Caco permeability [10^–6^ cm/s]/efflux factor	62/1.5	52/1.2	0.5/31	<1.1
Microsomal stability mouse/human [%QH]	50/48	73/75	<23/<23	<23/<23
Stability in human hepatocytes [%QH]	28	n.d.	10	n.d.

BI01383298 (6) inhibits human NaCT-mediated citrate uptake in endogenous and recombinant cell lines. Using a stable HEK293-Flp-in-hNaCT cell line expressing human NaCT, we could demonstrate that BI01383298 (6) and BI01455810 (8) inhibit human NaCT-dependent citrate uptake in a dose-dependent manner (IC_50_ = 50 nM and 285 nM, respectively; Table 1, Figure 5). As the immortalized human liver cell line HepG2 endogenously expresses human NaCT, we also tested the ability of the two compounds to inhibit citrate uptake in human HepG2 cells. We observed similar potency levels in comparison to the recombinant yet stably expressed NaCT with an observed IC_50_ of 24.5 nM and 184 nM, respectively (Table 1, Figure 5). The potency of BI01383298 (6) on human NaCT in our assay is similar to that quoted elsewhere in the literature^39^ and much improved over PF-06649298 (1).^12^ There was no detectable inhibition of citrate transport in either cell line when using the structural analog BI01372674 (7).

**Figure 5:**
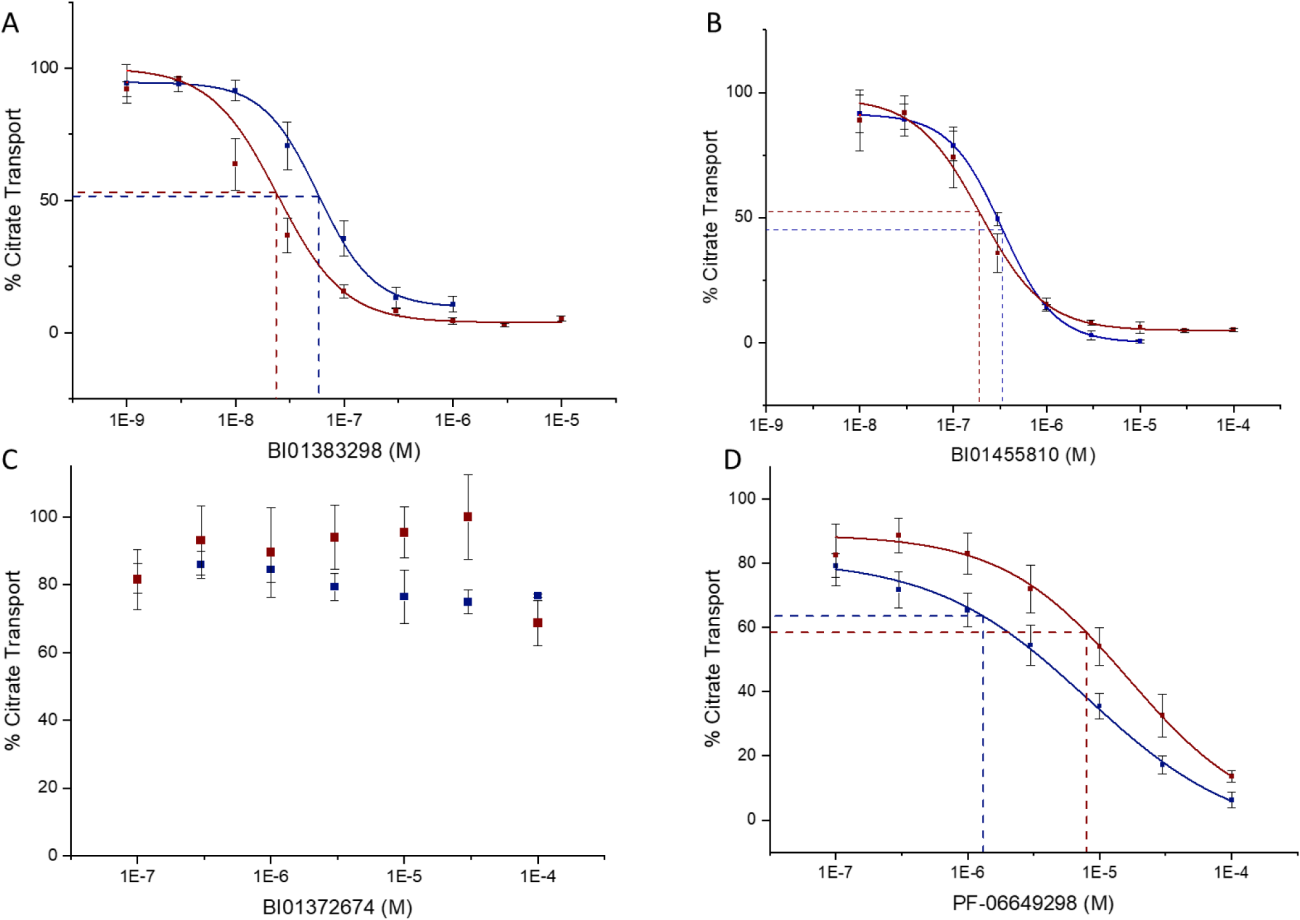
Inhibition of ^14^C-citrate uptake for compounds (A) BI01383298 (6), (B) BI01455810 (8), (C) BI01372674 (7), and (D) PF-06649298 (1). Data in blue show inhibition response to increasing compound concentrations in HEK293 cells recombinantly expressing NaCT (final ^14^C-citrate concentration: 2 μM/well). Data in brown show the inhibition response in an endogenous model (HepG2 cells) with a final ^14^C-citrate concentration of 1.8 μM/well with increasing concentrations of the given ligand. Corresponding IC_50_ values are presented in Table 1 from 6 to 9 repeats of 2–3 independent replicates.

In a head-to-head comparison, BI01383298 (6) proved to be more than 100-fold more potent than the citrate analog PF-06649298 (1) (Table 1). A synopsis of all potency data as well as basic *in vitro* pharmacokinetic data is given in Table 1. This includes solubility, parallel artificial membrane permeability assays (PAMPA), Caco-2 permeability, and microsomal stability data for BI01383298 (6), BI01455810 (8), BI01372674 (7), and the citrate analog PF-06649298 (1). Stability in human hepatocytes was determined for the two active piperidinecarboxamides 6 and 8. Although BI01383298 (6) solubility is low, it is still sufficient for delivery into cell-based assays. The permeability of BI01383298 (6) and BI01455810 (8) as measured by PAMPA and Caco-2 permeability assays are similar and significantly higher than for PF-06649298 (1). Metabolic clearance as assessed by microsomal stability in mice and human microsomes is higher for the two active piperidinecarboxamides (6 and 8) compared to PF-06649298 (1).

### BI01383298 (6) Is a Specific Inhibitor of hNaCT

To broaden the insights into the selectivity profile of BI01383298 (6), the inhibitory activity of this molecule was also tested using stable HEK293-Flp-in cell lines that express either human NaDC-1 or NaDC-2, the most closely related family members of the sodium-coupled transporter family. These related transporters also transport citrate. Making use of the Flp-in system, NaDC-1 and NaDC-2 cell lines have been generated using the same integration site and carry only a single copy of the respective transporter gene per cell. Detectable citrate transport activity was observed only in HepG2 cells. The integration of exogenous NaCT or homologous transporters into HEK293 cells was necessary to be able to make specific measurements of respective uptake activities.

In sharp contrast to the citrate analog PF-06649298 (1), the piperidinecarboxamide BI01383298 (6) showed no detectable inhibition of citrate uptake mediated by human NaDC-1 or NaDC-2. Surprisingly, BI01383298 (6) and BI01455810 (8) demonstrated a human-selective inhibition profile (mNaCT, IC_50_ > 70 μM, Table 1). A human-selective inhibition profile has also been observed by others when using up to 10 μM BI01383298 (6).^39,42^ This indicates that caution should be taken with BI01383298 (6) when using murine pharmacological models. The species selectivity of BI01383298 (6) contrasts with the recently reported nonsubstrate analogous NaCT inhibitor ETG-5773 (3), which inhibits both murine and human NaCT.^37^ Confirmation of the unexpectedly high degree of selectivity against murine NaCT (and hGlyT2) with the more soluble analog BI01455810 (8) allowed us to rule out a potential artifact based on the lower aqueous solubility of BI01383298 (6) (Table 1). In addition, both BI01383298 (6) and BI01455810 (8) did not inhibit hGlyT2, an unrelated transporter mediating glycine uptake.

Interestingly, others have reported that BI01383298 (6) acts in a way consistent with irreversible inhibition of NaCT.^39^ Based upon the chemical structure of BI01383298 (6), it seems unlikely that this compound would be able to act as a covalent inhibitor. For confirmation, we investigated BI01383298 (6) and a set of close analogs for solution stability with and without glutathione added. These experiments provided no indication for the covalent reactivity of BI01383298 (6). We conclude that slow binding kinetics to NaCT might be a more plausible explanation for the apparent irreversible behavior of BI01383298 (6). The reported failure to wash out the compound could be related to a combination of slow off-rate and partitioning into membranes, resulting in incomplete extraction even upon washing with serum-containing media.

Screening of BI01383298 (6) against a broad panel of 44 unrelated targets (Cerep SafetyScreen44)^43^ confirmed this compound’s high overall selectivity. Inhibition data at a compound concentration of 10 μM indicated more than 100-fold selectivity of BI01383298 (6) for 42 out of 44 targets tested. Only two targets, the rat kappa opioid receptor and the human CB1 receptor, demonstrated appreciable inhibition at 10 μM of 81% and 78%, respectively (see Table S2).

### The Piperidinecarboxamide BI01383298 (6) Directly Interacts with Human NaCT

To confirm target engagement, selected compounds from the piperidinecarboxamide class were tested using nano Differential Scanning Fluorimetry (nanoDSF) at concentrations between 0.1 and 10 μM. The greatest thermostabilization of human NaCT was observed for BI01383298 (6) (11.6 ± 1.0 °C), followed by BI01455810 (8) (7.4 ± 1.1 °C), and correlated well with the citrate uptake assays. PF-06649298 (1),^12^ a previously identified inhibitor of human NaCT, also stabilized human NaCT (8.8 °C ± 0.5 °C), as has previously been observed by a similar method.^36^

To confirm that the observed thermostabilization of NaCT was a result of a direct interaction with BI01383298 (6), a follow-up assay was performed at a range of ligand concentrations. The more soluble active analog BI01455810 (8) identified in the cell transport assay and initial DSF screen, as well as the structurally closely related negative control BI01372674 (7), were also tested. A dose-dependent response (Figure 6) was observed for BI01383298 (6) and BI01455810 (8). The negative control compound, BI01372674 (7), was not observed to stabilize hNaCT at any concentration tested. Both BI01383298 (6) and BI01455810 (8) had no effect on the thermostability of vcINDY (see Supplementary Table S3). The inability of these compounds to stabilize vcINDY is not surprising considering (i) the differences in the structure and transport mechanism of vcINDY^44^ and (ii) the high selectivity of this compound class already observed.

**Figure 6:**
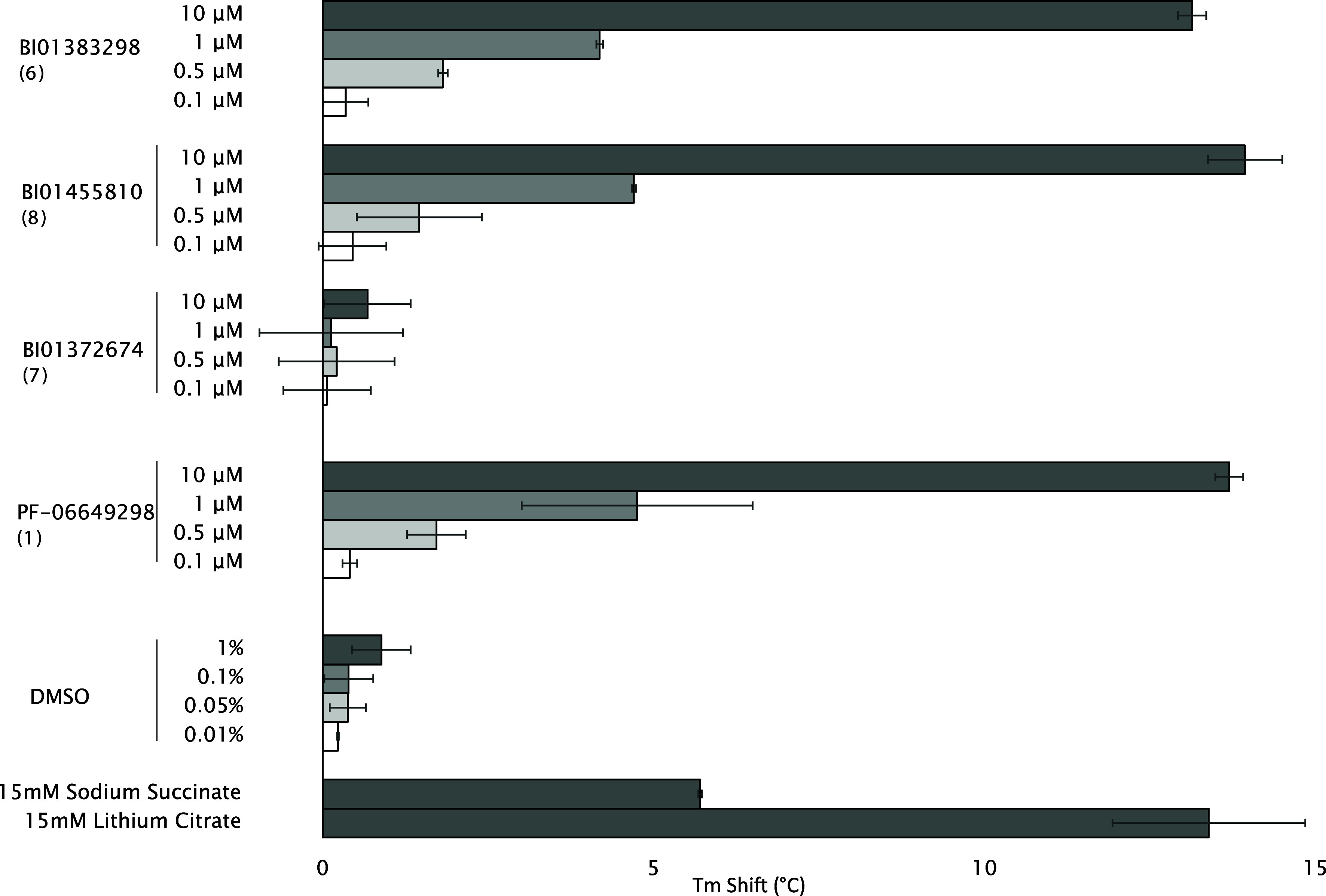
Target engagement by thermostability for selected compounds and the negative control. Compounds were added at a ratio of (compound:protein) of 10:1, 1:1, 1:2, and 1:10. Stabilization of NaCT was observed at a 1:2 ratio and higher above the background of the DMSO-matched control. Sodium succinate and lithium citrate were included as substrate-based controls at 50 mM.

### Impact of NaCT Inhibition by BI01383298 (6) and BI01455810 (8) on Citrate Metabolism

Using the Seahorse XF Extracellular Flux Analyzer, the impact of NaCT inhibition on cellular citrate metabolism was investigated using the stable HEK293-Flp-in-hNaCT cell line expressing human NaCT (Figure 7).

**Figure 7:**
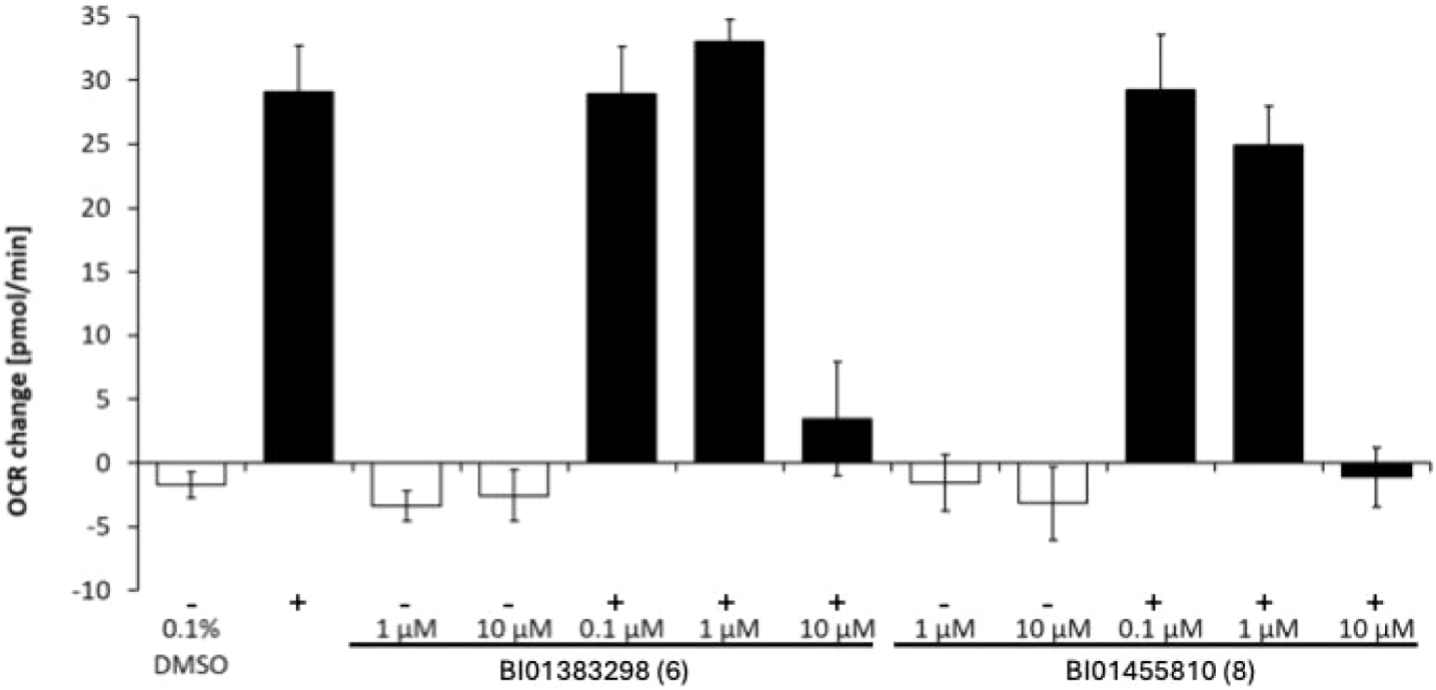
Change in the oxygen consumption rate (OCR) for HEK293-Flp-in-hNaCT cells in the presence (+) and absence (−) of 150 μM citrate after treatment with different concentrations of BI01383298 (6) and BI01455810 (8). OCR measures were repeated four to six times in a 2–1–3 min mix-wait-measure cycle on two biological replicates.

Similar to results reported previously, the addition of 150 μM citrate alone increased the cellular oxygen consumption rate (OCR) pronouncedly in the presence of NaCT (no effect using the control cell line; data not shown).^12^ The addition of the hNaCT inhibitor BI01383298 (6) alone at different concentrations did not alter the cellular OCR.

In the presence of citrate using stable HEK293-Flp-in-hNaCT cells, BI01383298 (6) was able to inhibit citrate-induced and hNaCT-mediated increases in the level of the OCR in a dose-dependent manner, demonstrating its impact on citrate-induced mitochondrial metabolism. To control for the functionality of the cells investigated and to exclude potential toxicities induced by the different treatment regimens, carbonyl cyanide-ρ-trifluoromethoxyphenylhydrazone (FCCP) was added at the end of the study to measure spare respiratory capacity. FCCP addition resulted in a similar OCR increase in all cell lines and in all treatment groups. Interestingly, we observe a similar response for BI01383298 (6) and BI01455810 (8). The improved solubility and overall physicochemical properties of BI01455810 (8) thus may contribute to its comparable cellular effect, despite its lower biochemical potency.

## Conclusion

We describe the discovery and characterization of a new class of inhibitors against the human sodium-coupled carboxylic acid transporter NaCT. The piperidinecarboxamide BI01383298 (6) is a potent and specific inhibitor of human NaCT with no inhibitor effect observed for other NaCT family members as well as murine NaCT. This pronounced species selectivity distinguishes BI01383298 (6) from the recently reported nonsubstrate analog NaCT inhibitor ETG-5773 (3).^37^ We therefore speculate that these two compounds address different binding sites on NaCT. While no panel selectivity data are reported for ETG-5773 (3), BI01383298 (6) is also highly selective against a standard panel of unrelated targets, demonstrating more than 100-fold selectivity against 42 of the panel and 10-fold selectivity against the remaining two. BI01383298 (6) is 100-fold more potent compared to the previously reported substrate analog inhibitor PF-06649298 (1).^12^

We also describe two additional tool compounds: a negative control compound, BI01372674 (7), and BI01455810 (8), a slightly less potent but more soluble inhibitor of NaCT. BI01372674 (7) is a close structural analog of BI01383298 (6) but does not inhibit NaCT. BI01455810 (8) shares the same scaffold but is more than 50-fold more soluble than BI01283298 (6). Despite being less potent, we also report on BI01455810 (8) here, as due to its improved *in vitro* ADME profile, it may be a more useful tool compound than BI01383298 (8). This set of tool compounds can be used to probe the function of NaCT, especially with respect to its suitability as a target for obesity and MAFLD and for rare diseases associated with NaCT mutations. Despite the predicted structural and sequence differences between murine and human NaCT, we did not expect to find such a large difference in the potency of BI01383298 (6) against murine and human NaCT. Due to this observed species selectivity, BI01383298 (6) and BI01455810 (8) are not suited for *in vivo* experiments with wild-type murine models. It remains an open question whether the piperidinecarboxamide class of NaCT inhibitors can be optimized to achieve activity in rodents, which would enable the development of *in vivo* tool compounds. Higuchi *et al.* ^39^ proposed a binding site for BI01383298 (6) based on docking studies using a humanized model of vcINDY. Our own analysis, using Boltz2 ^47^ and the human NaCT sequence, similarly places BI01383298 (6) within the citrate-binding pocket (Figure S4), consistent with the model reported by Higuchi *et al.* ^39^ However, the predicted binding affinities for human and mouse NaCT fail to account for the complete lack of activity of BI01383298 (6) on the mouse transporter. An experimental costructure of a representative compound from this series bound to NaCT will therefore be crucial for enabling further optimization of this. BI01383298 (6) and BI01372674 (7) can be ordered for free from Boehringer Ingelheim’s open science portal, opnMe.^38^

Given that rare brain diseases such as epilepsy have been associated with inactivating mutations in NaCT and the negative consequences of inhibiting NaCT in the brain, suggested by the link between epilepsy and NaCT inactivating mutations,^27^ efforts should now focus on developing small molecules that do not pass the blood–brain barrier or are targeted to hepatocytes. Future work on piperidinecarboxamide NaCT inhibitors to generate rodent-active *in vivo* tool compounds should therefore include low brain exposure as an optimization parameter to avoid CNS-mediated side effects.

## Experimental Section

### Materials

All solvents and reagents were obtained from commercial sources and were used as received. Reactions were carried out in conventional glassware without the application of inert gas. Reactions were monitored by an HPLC-MS analysis. Unless stated otherwise, evaporations of reaction mixtures or product solutions as well as coevaporations were performed using a rotary evaporator under reduced pressure, applying a water bath (temperature 25–50 °C). Unless stated otherwise, crude products were purified by flash column chromatography on silica (using a Biotage IsoleraOne, Biotage IsoleraFour, or CombiFlash Teledyne Isco system) or by (semi)­preparative reversed-phase HPLC (Agilent Acquity or Waters instruments). Nuclear magnetic resonance (NMR) spectra were recorded at temperature 303 K (30 ± 1 °C) on a Bruker Avance HDIII 400 spectrometer in deuterated DMSO-*d*_6_ using tetramethylsilane as an internal reference. Chemical shifts δ are reported in parts per million (ppm). ^1^H NMR spectra were referenced to the residual partially nondeuterated solvent signal of DMSO (δ = 2.50 ppm). Coupling constants *J* are reported in Hz, and splitting patterns are described as br = broad, s = singlet, d = doublet, t = triplet, q = quartet, quin = quintet, and m = multiplet. Purity of all compounds (determined by ^1^H-NMR) is ≥95%. In addition, the reported probe compounds and the negative control (6–8) were confirmed to be >95% pure by HPLC analysis.

#### 1-(3,5-Dichlorobenzenesulfonyl)­piperidine-4-carboxylic Acid Methyl Ester (**19**)

To a solution of 10.0 g (40.7 mmol) 3,5-dichlorobenzenesulfonyl chloride in 100 mL of dichloromethane (DCM) were added 6.05 mL (44.8 mmol) piperidine-4-carboxylic acid methyl ester and 6.25 mL (44.8 mmol) triethylamine. The mixture was stirred overnight at ambient temperature and then washed with aqueous hydrochloric acid (1 mol/L) and water. The organic layer was dried over magnesium sulfate, filtered, and evaporated to dryness to yield a colorless solid (14.9 g), which was taken to the next step without further purification. ^1^H NMR (400 MHz, DMSO-*d*_6_) δ ppm 8.04 (t, *J* = 1.9 Hz, 1H), 7.75 (d, *J* = 1.9 Hz, 2H), 3.62–3.56 (m, 2H), 3.59 (s, 3H), 2.56–2.40 (m, 3H), 1.95–1.87 (m, 2H), 1.61–1.40 (m, 2H). MS ESI: (M + H)^+^ 352.

#### 1-(3,5-Dichlorobenzenesulfonyl)­piperidine-4-carboxylic Acid (**20**)

14.9 g of 1-(3,5-dichlorobenzenesulfonyl)­piperidine-4-carboxylic acid methyl ester (**19**) (crude product taken from the previous step) was dissolved in a mixture of 31.7 mL of aqueous NaOH (4 mol/L, 127 mmol) and 100 mL of methanol. The mixture was stirred at 70 °C for 3 h and then cooled to ambient temperature. Aqueous hydrochloric acid (4 mol/L) was added with stirring. Upon addition of further water, the precipitate formed was filtered off with suction and dried under vacuum at 50 °C to yield a colorless solid (12.9 g; 38.1 mmol, 94% over two steps). ^1^H NMR (400 MHz, DMSO-*d*_6_) δ ppm 12.30 (br s, 1H), 8.03 (t, *J* = 1.9 Hz, 1H), 7.75 (d, *J* = 1.9 Hz, 2H), 3.59–3.51 (m, 2H), 2.58–2.48 (m, 2H), 2.36–2.27 (m, 1H), 1.93–1.85 (m, 2H), 1.60–1.48 (m, 2H). MS ESI: (M – H)^−^ 336, (M + H)^+^ 338.

#### 1-(3,5-Dichlorobenzenesulfonyl)­piperidine-4-carboxylic Acid 4-Fluorobenzylamide (**6**, **BI01383298**)

700 mg (2.07 mmol) of 1-(3,5-Dichlorobenzenesulfonyl)-piperidine-4-carboxylic acid (**20**), 235 μL (2.07 mmol) of 4-fluorobenzylamine, 665 mg (2.07 mmol) of *O*-(benzotriazol-1-yl)-*N*,*N*,*N*′,*N*′-tetramethyluronium tetrafluoroborate (TBTU), and 288 μL (2.07 mmol) of triethylamine were dissolved in 10 mL of DMF. The mixture was stirred overnight at ambient temperature, and then ice–water was added with stirring. The precipitate formed was filtered off with suction, washed with water, and dried *in vacuo* at 50 °C. The crude product was recrystallized from 100 mL of methanol, filtered off at 0 °C, washed with cold methanol, and dried *in vacuo* at 50 °C to yield 794 mg (1.78 mmol, 86%) of colorless flocculent crystals. ^1^H and ^13^C NMR spectra and an HPLC chromatogram for compound **6** can be found in Figures S3, S4, and S5, respectively. ^1^H NMR (400 MHz, DMSO-*d*_6_) δ ppm 8.27 (t, *J* = 6.0 Hz, 1 H) 8.02 (t, *J* = 1.9 Hz, 1 H) 7.75 (d, *J* = 1.9 Hz, 2 H) 7.19–7.28 (m, 2 H) 7.07–7.15 (m, 2 H) 4.21 (d, *J* = 5.9 Hz, 2 H) 3.65 (dt, *J*12.0, 3.2 Hz, 2 H) 2.48 (td, *J* = 11.9, 2.7 Hz, 2 H) 2.22 (tt, *J* = 11.1, 3.9 Hz, 1 H) 1.80 (br dq, *J* = 13.5, 3.3 Hz, 2 H) 1.58 (dtd, *J* = 13.5, 11.1, 11.1, 4.1 Hz, 2 H) ^13^C NMR (101 MHz, DMSO-*d*_6_) δ ppm 173.3 (s) 161.0 (d, *J* = 242.2 Hz) 139.2 (s) 135.7 (d, *J* = 2.6 Hz) 135.3 (s) 132.7 (s) 128.9 (d, *J* = 8.2 Hz) 125.7 (s) 114.9 (d, *J* = 21.1 Hz) 45.2 (s) 41.2 (s) 27.6 (s). MS ESI/APCI: (M + H)^+^ 445, (M + NH_4_)^+^ 462, (M – H)^−^ 443, and (M + HCOO)^−^ 489.

#### 4-(Dimethylphosphinoyl)­benzonitrile (**21**)

A mixture of 10.0 g (43.7 mmol) of 4-iodobenzonitrile, 3.58 g (45.8 mmol) of dimethylphosphinoxide, 18.5 g (56.8 mmol) of cesium carbonate, and 100 mL of MeCN was degassed by bubbling through argon. 1.26 g (2.18 mmol) of 4,5-bis­(diphenylphosphino)-9,9-dimethylxanthene (xantphos) and 1.00 g (1.09 mmol) of tris­(dibenzylideneacetone) dipalladium were added, and the mixture was stirred under argon overnight at 80 °C. Water was added, and the mixture was extracted with ethyl acetate. The organic layer was separated, dried with magnesium sulfate, filtered, and evaporated. The crude product was purified by silica gel chromatography (DCM/methanol, linear gradient 0–15% methanol) to yield 1.48 g (8.26 mmol, 19%) of a light brown powder. ^1^H NMR (400 MHz, DMSO-*d*_6_) δ ppm 8.02–7.94 (m, 4H), 1.70 (d, *J* = 13.4 Hz, 6H). MS ESI: (M + H)^+^ 180.

#### 4-(Dimethylphosphinoyl)­benzylamine (**22**)

To a solution of 2.06 g (11.5 mmol) of 4-(Dimethylphosphinoyl)­benzonitrile (**21**) in 20 mL of methanolic ammonia were added 200 mg of Raney nickel. The mixture was stirred under 50 psi of hydrogen pressure at ambient temperature overnight. The catalyst was filtered off with suction, and the filtrate was evaporated to yield the crude product (2.23 g) that was taken to the next step without further purification. ^1^H NMR (400 MHz, DMSO-*d*_6_) δ ppm 7.76–7.63 (m, 2 H), 7.52–7.42 (m, 2 H), 3.85–3.69 (m, 2 H), 1.62 (d, *J* = 13.1 Hz, 6 H). MS ESI: (M + H)^+^ 184.

#### 1-(3,5-Dichlorobenzenesulfonyl)­piperidine-4-carboxylic Acid 4-(Dimethylphosphinoyl)­benzylamide (**7**, **BI01372674**)

600 mg (1.77 mmol) of 1-(3,5-dichlorobenzenesulfonyl)­piperidine-4-carboxylic acid (**20**), 325 mg of crude 4-dimethylphosphinoylbenzylamine (**22**) from the previous step, 570 mg (1.77 mmol) of TBTU, and 496 μL (3.55 mmol) of trimethylamine were dissolved in 3 mL of DMF. The mixture was stirred overnight at ambient temperature. Further 325 mg (1.77 mmol) of 4-dimethylphosphinoylbenzylamine, 570 mg (1.77 mmol) of TBTU, and 496 μL (3.55 mmol) of triethylamine were added, and the mixture was stirred for a further 3 h. Ice–water was added, and the mixture was extracted with ethyl acetate. The organic layer was separated, dried with magnesium sulfate, filtered, and evaporated. The crude residue was purified by preparative RP-HPLC using a C18 column (Waters XBridge, water/MeCN/aq. ammonia), yielding 370 mg (735 mmol, 41%) of a colorless solid. ^1^H and ^13^C NMR spectra and an HPLC chromatogram for compound **7** can be found in Figures S6, S7, and S8, respectively. ^1^H NMR (400 MHz, DMSO-*d*_6_) δ ppm 8.34 (t, *J* = 5.9 Hz, 1H) 8.03 (t, *J* = 1.9 Hz, 1H) 7.76 (d, *J* = 1.9 Hz, 2H) 7.65–7.73 (m, 2H) 7.29–7.37 (m, 2H) 4.28 (d, *J* = 5.9 Hz, 2H) 3.66 (dt, *J* = 12.0, 3.2 Hz, 2H) 2.48 (td, *J* = 11.8, 2.7 Hz, 2H) 2.24 (tt, *J* = 11.2, 3.8 Hz, 1H) 1.82 (br dq, *J* = 13.5, 3.1 Hz, 2H) 1.62 (d, *J* = 13.3 Hz, 6H) 1.53–1.65 (m, 2H). ^13^C NMR (101 MHz, DMSO-*d*_6_) δ ppm 173.4 (s) 143.0 (d, *J* = 2.6 Hz) 139.2 (s) 135.3 (s) 134.3 (br d, *J* = 96.4 Hz) 132.7 (s) 129.7 (d, *J* = 10.3 Hz) 127.0 (d, *J* = 11.6 Hz) 125.8 (s) 45.2 (s) 41.7 (s) 40.1 (s) 27.7 (s) 17.7 (d, *J* = 70.6 Hz). MS ESI/APCI: (M + H)^+^ 503, (M – H)^−^ 501.

#### 1-(3-Bromo-5-methylbenzenesulfonyl)­piperidine-4-carboxylic Acid Methyl Ester (**23**)

To a solution of 1.00 g (3.71 mmol) of 3-bromo-5-methylbenzenesulfonyl chloride in 20 mL of DCM was added 551 μL (4.08 mmol) of piperidine-4-carboxylic acid methyl ester and 569 μL (4.08 mmol) of triethylamine. The mixture was stirred overnight at ambient temperature, then washed with aqueous hydrochloric acid (1 mol/L) and water. The organic layer was dried over sodium sulfate, filtered, and evaporated to dryness to yield a colorless oil (1.42 g) that crystallized upon standing and was taken to the next step without further purification. ^1^H NMR (400 MHz, DMSO-*d*_6_) δ ppm 7.78 (br s, 1H), 7.65 (br s, 1H), 7.57 (br s, 1H), 3.61–3.54 (m, 2H), 3.58 (s, 3H), 2.52–2.38 (m, 3H), 2.41 (s, 3H), 1.95–1.87 (m, 2H), 1.62–1.50 (m, 2H). MS ESI: (M + H)^+^ 376.

#### 1-(3-Bromo-5-methylbenzenesulfonyl)­piperidine-4-carboxylic Acid (**24**)

1.40 g (3.72 mmol) of 1-(3-bromo-5-methylbenzenesulfonyl)­piperidine-4-carboxylic acid methyl ester (**23**) was dissolved in a mixture of 2.79 mL of aqueous NaOH (4 mol/L, 11.2 mmol) and 10 mL of methanol. The mixture was stirred at 70 °C for 3 h, then cooled down to ambient temperature. Aqueous hydrochloric acid (4 mol/L) was added with stirring. Upon addition of further water, the precipitate formed was filtered off with suction and dried *in vacuo* at 50 °C to yield a colorless solid (1.16 g; 3.20 mmol, 86%). ^1^H NMR (400 MHz, DMSO-*d*_6_) δ ppm 12.29 (br s, 1H), 7.78 (br s, 1H), 7.64 (br s, 1H), 7.57 (br s, 1 H), 3.53–3.45 (m, 2H), 2.51–2.42 (m, 2H), 2.41 (s, 3H), 2.34–2.24 (m, 1H), 1.92–1.84 (m, 2H), 1.60–1.49 (m, 2H). MS ESI: (M – H)^−^ 360, (M + H)^+^ 362.

#### 1-(3-Bromo-5-methylbenzenesulfonyl)­piperidine-4-carboxylic Acid (Pyridin-2-ylmethyl)­amide (**8**, **BI01455810**)

A solution of 150 mg (0.414 mmol) of 1-(3-bromo-5-methylbenzenesulfonyl)­piperidine-4-carboxylic acid (**24**), 146 mg (0.455 mmol) of TBTU, and 172 μL (1.24 mmol) of triethylamine in 10 mL of DMF was stirred at ambient temperature for 10 min. 47 μL (0.455 mmol) of 2-(aminomethyl)­pyridine was added, and the mixture was stirred for 4 h at ambient temperature. The mixture was poured on ice–water and stirred until the ice had melted. The precipitate was filtered off with suction, washed with water, and dried *in vacuo* at 50 °C to yield 175 mg of crude product as a colorless powder. Further purification by preparative RP-HPLC using a C18 column (Waters XBridge, water/MeCN/aq. ammonia) yielded 157 mg (0.347 mmol, 84%) of a colorless solid. ^1^H and ^13^C NMR spectra for compound **8** can be found in Figures S9 and S10. ^1^H NMR (400 MHz, DMSO-*d*_6_) δ ppm 8.41–8.52 (m, 1H) 8.32 (t, *J* = 5.9 Hz, 1H) 7.78 (ddd, *J* = 1.8, 1.5, 0.7 Hz, 1H) 7.73 (td, *J* = 7.7, 1.8 Hz, 1H) 7.66 (td, *J* = 1.7, 0.6 Hz, 1H) 7.58 (td, *J* = 1.5, 0.8 Hz, 1H) 7.24 (ddd, *J* = 7.5, 4.8, 1.2 Hz, 1H) 7.20 (dt, *J* = 7.8, 1.0 Hz, 1H) 4.32 (d, *J* = 5.9 Hz, 2H) 3.62 (dt, *J* = 12.0, 3.2 Hz, 2H) 2.42 (q, *J* = 0.5 Hz, 3H) 2.40 (td, *J* = 11.7, 2.7 Hz, 2H) 2.26 (tt, *J* = 11.2, 3.8 Hz, 1H) 1.83 (br dq, *J* = 13.7, 3.0 Hz, 2H) 1.61 (dtd, *J* = 13.7, 11.2, 11.2, 4.1 Hz, 2H). ^13^C NMR (101 MHz, DMSO-*d*_6_) δ ppm 173.5 (s) 158.6 (s) 148.7 (s) 142.1 (s) 137.5 (s) 136.6 (s) 136.2 (s) 126.7 (s) 126.6 (s) 122.0 (s) 121.9 (s) 120.7 (s) 45.3 (s) 44.0 (s) 40.1 (s) 27.7 (s) 20.4 (s). MS ESI/APCI: (M + H)^+^ 452; (M – H)^−^ 450; (M + HCOO)^−^ 496.

### Cloning and Expression of NaCT

The gene for NaCT (SLC13A5) was purchased from the Mammalian Gene Collection, IMAGE:8143798. NaCT was expressed using a construct consisting of the full-length gene with a C-terminal purification tag comprising a tobacco etch virus (TEV) protease cleavage site, a 10xHis purification sequence, and a FLAG tag in the expression vector pFB-CT10HF-LIC (available from the Structural Genomics Consortium (SGC)). Baculoviruses were produced by the transformation of DH10Bac cells. *Spodoptera frugiperda* (Sf9) insect cells in Sf-900 II SFM medium (Life Technologies) were split to densities between 1 × 10^6^ and 2 × 10^6^ 24 h before infection. After infection with recombinant baculovirus, cells were incubated for 72 h at 27 °C in 3 L capacity glass shaker flasks.

### Selection of Suitable Detergents for NaCT Purification

We assessed the size and composition of the protein-detergent complex formed by NaCT over a range of detergents. We combined size exclusion chromatography (SEC) with a Sepax SRT 300 column on a Dionex micro-HPLC system. Purifications were performed as described below with 1% detergent (w/v) ± 0.1% cholesteryl hemisuccinate (CHS) during protein solubilization and a concentration of 3× the detergent critical micelle concentration (CMC, Anatrace) ± CHS (10:1 detergent:CHS) in all other purification buffers.

### Production of SLC13A5 for *In Vitro* Studies

Between 12 L (infection density 2 × 10^6^ cells/mL) and 24 L (infection density 2–4 × 10^6^ cells/mL) of recombinant cell culture were processed for each purification. For each liter of insect cell culture equivalent (at 2 × 10^6^ cells/mL) cells were resuspended in 50 mL of lysis buffer (50 mM HEPES, pH 7.5, 200 mM NaCl, 1 Roche protease inhibitor cocktail) and lysed by two passes through an EmulsiFlex-C5 homogenizer (Aventis). Protein was extracted from cell membranes by incubation of the crude lysate with 1% DDM for 1 h at 4 °C. Cell debris and unlysed cells were removed by centrifugation at 35,000g for 1 h. Detergent-solubilized protein was purified by immobilized metal affinity chromatography by batch binding to 1 mL of 50% Co^2+^-charged TALON resin (Clontech) at 4 °C for 1 h. The resin was washed with 20 column volumes of wash buffer (50 mM HEPES, pH 7.5, 200 mM NaCl, and 20 mM imidazole with 0.03% DDM) and eluted with wash buffer supplemented with 250 mM imidazole. Imidazole was immediately removed using a PD10 column (GE Healthcare Life Sciences) and an elution buffer lacking imidazole and glycerol. The PD10-eluted protein was treated with 20:1 (w:w, protein:protease) TEV protease overnight at 4 °C. The TEV protease-cleaved protein was separated from the 6xHis-tagged TEV protease and uncleaved NaCT by incubation for 1 h with TALON resin at 4 °C. The resin was collected in a column, and the flow-through and initial wash with SEC buffer were concentrated in a 100 kDa cutoff, PES, 2 mL Corning concentrator and further purified by size exclusion chromatography (SEC) using a Superose 6 Increase 10/300GL column (GE Healthcare Life Sciences) equilibrated with SEC buffer (20 mM HEPES, pH 7.5, 200 mM NaCl, 0.015% DDM) collected and concentrated to 2–3 mg/mL using a 100 kDa cutoff PES Sartorius concentrator. The molecular weight of each purified SLC13A5 construct was confirmed using an MSD-ToF electrospray ionization orthogonal time-of-flight mass spectrometer (Agilent Technologies Inc., Palo Alto, CA, USA).^45^

### Membrane Potential Assays

Using the FLIPR Membrane Potential Kit for measuring membrane potential (Molecular Devices, catalog no. R-8123), compounds from the Boehringer Ingelheim compound collection were screened at a concentration of 10 μM. Frozen HEK293-Flp-in cells expressing either human NaCT or human SGLT2 (typically 1 × 10^8^ cells/vial) were revived in a 37 °C water bath and resuspended in 40 mL of assay medium (DMEM, supplemented with 10% FCS, 1% Pen/Strep, and 400 μg/mL Geneticin/G418). The cell suspension was centrifuged at 153 × *g* for 5 min and resuspended in 40 mL assay medium. The cell suspension was subsequently diluted to a concentration of 0.625 × 10^6^ cells/mL in assay medium and then aliquoted into assay plates (sterile BD PCA 384-well plates; 40 μL/well). Cells were incubated for 24 h at 37 °C in the presence of 5% CO_2_ at 95% relative humidity. The following day, each well was washed twice with 90 μL of assay buffer at pH 7.4 (10 mM HEPES, 140 mM NaCl, 5.4 mM KCl, 1 mM CaCl_2_, 0.8 mM MgSO_4_, 0.4 mM KH_2_PO_4_, 0.4 mM NaH_2_PO_4_, 25 mM glucose). After the last wash, 20 μL of buffer was left in each well. Ten μL of membrane potential dye solution (dissolved in 50 mL of assay buffer) was added. Assay plates were incubated for 360 min at 37 ± 1 °C in the presence of 5% CO_2_ at 95% relative humidity. Ten μL per well of each test compound solution in assay buffer, containing 5% DMSO, was added (final test compound concentration 10 μM), and the assay plates were incubated for 15 min at room temperature. Ten μL of well stimulation buffer (assay buffer containing 4.45 mM citric acid, pH-adjusted to pH 7.4 by addition of KOH), or assay buffer only, was added in parallel to measure fluorescence emission using the FLIPR instrument.

### Preparation of Compounds for Citrate Uptake Assays

Test compound dilutions (2-fold concentrated); starting from 10 mM stock solutions in 100% DMSO, compounds were diluted in pure DMSO using the appropriate dilution steps. Prior to the uptake assay, 200 μL (Flp-in) or 400 μL (HepG2) assay medium + 4.8 μL compound solution (or pure DMSO for noninhibited control wells) were mixed (final DMSO concentration in the assay: 1% (v/v)).

To obtain a 10 mM Li^+^ concentration in the test wells, 400 μL of assay medium + 4.8 μL of DMSO + 2.4 μL of LiCl stock solution (2 M LiCl dissolved in water) were mixed (final composition in the assay: 10 mM LiCl + 1% (v/v) DMSO).

### Preparation of Cells for Citrate Uptake Assays

HEK293-Flp-in cells were stably transfected with (A) human NaCT, (B) human NaDC1, (C) human NaDC2, and (D) murine NaCT, according to the manufacturer’s instructions. The HEK293-Flp-in host cell line was used to ensure a single integration of the transgene at a prespecified integration site. The final stable cell lines used in this study were HEK293-Flp-in-hNaCT, HEK293-Flp-in-hNaDC1, HEK293-Flp-in-hNaDC2, and murine HEK293-Flp-in-mNaCT. Cell lines were grown in DMEM + 10% FBS + 100 μg/mL Hygromycin using 75 cm^2^ cell culture flasks at 37 °C. Prior to uptake assays, the medium of the confluent cultures was discarded, and the cells were washed with DPBS. The cells were detached by the addition of 2.0 mL Accumax per 75 cm^2^ culture flask for up to 3 min at 37 °C and resuspended in 10 mL assay medium. Following cell counting, 50000 cells per well were seeded in a volume of 50 μL into the OptiPlates (→1 fold-concentrated test compound dilutions).

HepG2 cells were cultivated in 75 cm^2^ flasks using a medium consisting of EMEM + 10% FBS + 1× NEAA and 10 mM l-Glutamine. For the uptake assay, the medium of the confluent cultures was discarded, and the cells were washed with DPBS. Cells were detached by the addition of 1 mL of 2× Trypsin in DPBS with 7.5 mM EDTA per 75 cm^2^ culture flask for up to 3 min at 37 °C and resuspended in 10 mL of cell culture medium. Following cell counting, 100,000 cells/well were seeded in a volume of 200 μL per well into Cytostar-T Scintillating 96-well microplates. Intentionally, during the seeding process, a few wells are not seeded with cells to obtain a BLANK control value. After overnight incubation in a humid cell culture incubator at 37 °C and 5% CO_2_, the cell culture medium was carefully aspirated, and the cells were incubated with 200 μL of prewarmed (37 °C) assay medium per well for at least 1 h prior to the experiment.

### Citrate Uptake Assay

The uptake of ^14^C-citrate into HEK293-Flp-in cells was monitored using the WGA-PVT SPA beads that bind to the HEK293-Flp-in cells due to their WGA-PVT surface. ^14^C-citrate uptake into HepG2 cells was monitored by using Cytostar-T Scintillating 96-well microplates. This latter assay format is based on the imported radioactive citrate brought into proximity with the SPA beads or the scintillant contained within the base of the plate by virtue of the biological processes within the cells. The radioactive decay is converted into a light signal that can be measured using e.g., the TopCount plate reader.

For HEK293-Flp-in cells, the assay medium consisted of 1× HBSS (contains 5.56 mM glucose) + 19.44 mM glucose (final concentration of glucose is 25 mM). A ^14^C-citrate working solution was prepared (volumes required per 96-well). For Flp-in cells, 2.1 mL of assay medium was added to 23.65 μL of ^14^C-citric acid stock solution (stock: 0.1 mCi/mL = 111,000,000 dpm/500 μL; EK = 50,000 dpm/well). 50 μL of the 2-fold-concentrated test compound dilutions were pipetted into each well of the OptiPlates-96 (*n* = 3). Microplate lids-96 were placed on top, and plates were incubated at 37 °C for 20 min. After 20 min, 20 μL of ^14^C-citrate working solution was added to each well (final volume per well: 120 μL; final ^14^C-citrate concentration: approximately 2 μM/well). After incubation for 4 h at 37 °C in a humid cell culture incubator, 30 μL of WGA-PVT SPA beads were added to each well (0.25 mg/well), and the plates were sealed on the top using a transparent plastic foil. Plates were subsequently incubated for 1 h at room temperature and gentle shaking. After incubation, the plate was placed into the TopCount NXT HTS, and the signal recorded was recorded.

For HepG2 cells, the assay medium consisted of 50% DMEM + 50% Ham’s F-12 + 15 mM HEPES (pH 7.4) (medium contains 15.5 mM glucose). 1.88 mL assay medium was added to 21.2 μL ^14^C-citric acid stock solution (stock: 0.1 mCi/mL = 111,000,000 dpm/500 μL; 1.391 mmol/L). Immediately prior to the uptake assay, the assay medium is carefully removed, and 100 μL of test compound dilution is added to each well of the Cytostar-T Scintillating 96-well Microplate (*n* = 3). Following a 20 min incubation at 37 °C in the cell culture incubator, 20 μL of 14C-citrate working solution is added to each well (final volume per well: 120 μL; final ^14^C-citrate concentration ∼1.8 μM/well). After incubation overnight at 37 °C in the cell culture incubator, the plates were sealed on the top using a transparent plastic foil and, on the bottom, using white plastic foil. Following sealing, the plate was placed into the TopCount NXT HTS, and the signal was recorded using protocol RadioNuclid ^14^C-Microscint.

### Glycine Uptake Assay

The uptake of ^3^H-glycine into HEK293 cells overexpressing the human GlyT2 receptor was monitored using Cytostar-T Scintillating 96-well microplates. Final glycine concentration during assay incubation was 250 nM. This uptake assay was performed analogously to the citrate uptake assay described above with the following specifics: the human embryonic kidney 293 cell clone (HEK293-hGlyT2 #13) overexpressing the human GlyT2 transporter was cloned in-house (the plasmid pCMV6-XL5-hGlyT2 containing the cDNA coding for the human GlyT2 transporter was obtained from Origene; the cDNA for GlyT2 was taken from this plasmid and subcloned into pcDNA3.1zeo from Invitrogen). Cell culture medium: DMEM + Glutamax (cat. no. 31966 from Gibco) with 10% FBS and 100 μg/mL Zeocin (cat. no. 25001 from Invitrogen).

### Thermostability of Human NaCT by Nano Differential Fluorimetry

All assays were carried out using a Prometheus NT (Nanotemper GmbH) instrument with standard capillaries. NaCT was diluted to 2 μM in 50 mM HEPES (pH 7.5), 200 mM NaCl, and 0.03% DDM. NaCT was mixed in a one-to-one ratio with either assay buffer (control), compound (between 0.1 and 20 μM), substrate (30–100 mM), or salt (30–100 mM). Approximately 10 μL of sample was loaded into each capillary, with care taken to avoid wicking protein up the sides of the capillary. Samples were processed for three biological samples covering 4–8 replicates for each. An unfolding ramp was performed at 70–90% power between 20 and 94 °C with a temperature increase of 1 °C per minute. *T*_m_ values were calculated using the onboard software using the integrated fluorescence ratio (350 nm/330 nm).

### Thermal Stability Assay with vcINDY

The thermal stability of vcINDY was monitored by nanoDSF.^46^ Protein production for vcINDY was performed as described.^47^ Protein was measured at a concentration of 5 μM in gel filtration buffer (50 mM Tris, pH 7.5, 100 mM NaCl, 5% glycerol, and 0.15% N-decyl-β-maltoside) in standard grade nanoDSF capillaries (Nanotemper) in a Prometheus NT.48 device (Nanotemper) controlled by PR. ThermControl (version 2.1.2). Excitation power was adjusted to 30–50%, and samples were heated from 20 to 90 °C with a slope of 1 °C/min. All samples were run in duplicates. For the ligand screen, vcINDY in gel filtration buffer supplemented with 0.5% DMSO was chosen as a reference. Ligands were prepared as 50 mM DMSO stock solutions and added to a final concentration of 500 μM ligand (0.5% DMSO). Samples were incubated for at least 10 min at RT prior to analysis.

### Aqueous Solubility

The aqueous solubility of the test compounds was determined in a high-throughput setup by comparing the amount dissolved in buffer to the amount in an acetonitrile/water (1/1) solution. Ten μL of 10 mM DMSO stock solution aliquots are diluted in a ratio of 1:40 with three different buffers (pH 2.2, 4.5, and 6.8). After 24 h of shaking, the solutions were filtered and analyzed by LC-UV. The amount dissolved in the buffer was compared to the amount dissolved in the acetonitrile solution. In case the compound is fully solubilized, a value larger than the upper assay wall, which is 250 μmol/L, is reported. For very poorly soluble compounds, hitting the lower assay wall, a value of less than 1 μg/mL is reported.

### Passive Permeability in the Parallel Artificial Membrane Permeability Assay (PAMPA)

The PAMPA assay provides data on the passive permeability of test compounds through immobilized artificial phospholipid membranes. A sandwich is formed from a 96-well microtiter plate and a 96-well polycarbonate microfilter plate, such that each composite well is divided into two chambers: donor and acceptor, separated by a microfilter disc coated with structured layers of phospholipids (Corning BioCoat Precoated PAMPA Plate System, Product Number 353015). A compound solution is added to the donor side, and after a certain time of incubation, compound concentrations in the acceptor *C*_n_ well are measured by LC/MS and compared to the concentration in the donor compartment prior to incubation *C*_0_. From this information, the permeability coefficient (PE) through the artificial membrane is calculated. The assay is run with pH 7.4 at both the acceptor and donor side. The apparent permeability *P*_app_ [cm/s] is given by

$$P_{\mathrm{app}}=\frac{V_{\mathrm{rec}}\cdot C_{\mathrm{rec}}}{A\cdot C_{\mathrm{don}}\cdot t}$$

where A [cm^2^] is the area of the filter, *C*_don_ [μmol/mL] is the substance concentration in the donor compartment at time *t*_0_, *V*_rec_ [mL] is the volume of buffer in the receiver compartment, *C*_rec_ [μmol/mL] is the substance concentration in the receiver compartment at time *t*_n_, and *t* [s] is the incubation time.

### Drug Transport across Human Caco-2 Cells

The assay provides information on the potential of a compound to pass the cell membrane and on the extent of oral absorption, as well as on whether the compound is actively transported by uptake and/or efflux transporters. Permeability measurements across polarized, confluent Caco-2 cell monolayers grown on permeable filter supports are used as the *in vitro* absorption model.

Apparent permeability coefficients (PE) of the compounds across the Caco-2 monolayers are measured (pH 7.2, 37 °C) in apical-to-basal (AB) (absorptive) and basal-to-apical (BA) (secretory) transport directions. AB permeability (PEAB) represents drug absorption from the intestine into the blood, and BA permeability (PEBA) represents drug secretion from the blood back into the intestine via both passive permeability and active transport mechanisms mediated by efflux and uptake transporters that are expressed on the Caco-2 cells. The compounds are assigned to permeability/absorption classes by comparison of the AB permeabilities with the AB permeabilities of reference compounds with known *in vitro* permeability and oral absorption in the human. Identical or similar permeabilities in both transport directions indicate passive permeation, and vectorial permeability points to additional active transport mechanisms. Higher PEBA than PEAB suggests the involvement of an apical efflux transporter (like P-gp) and/or a basolateral uptake transporter; higher PEAB than PEBA permeability suggests the involvement of an apical uptake transporter (like PepT1) and/or a basolateral efflux transporter (like MRP3). Active transport is concentration-dependently saturable.

Caco-2 cells ((1–2) × 10^5^ cells/1 cm^2^ area) were seeded on filter inserts (Costar Transwell polycarbonate or PET filters, 0.4 μm pore size) and cultured (DMEM) for 10 to 25 days. Compounds were dissolved in the appropriate solvent (like DMSO, 1–20 mM stock solutions). Stock solutions were diluted with HTP-4 buffer (128.13 mM NaCl, 5.36 mM KCl, 1 mM MgSO_4_, 1.8 mM CaCl2, 4.17 mM NaHCO_3_, 1.19 mM Na_2_HPO_4_·7H_2_O, 0.41 mM NaH_2_PO_4_·H2O, 15 mM HEPES, 20 mM glucose, pH 7.2) containing 0.25% BSA to prepare the transport solutions (0.1–300 μM compound, final DMSO ≤ 0.5%). The transport solution (TL) was applied to the apical or basolateral donor side for measuring A-B or B-A permeability (3 filter replicates), respectively. The receiver side contained HTP-4 buffer supplemented with 0.25% BSA. Samples were collected from the donor side at the start and end of the experiment, and from the receiver side at various time intervals for up to 2 h to determine the concentration by HPLC-MS/MS or scintillation counting. Sampled receiver volumes were replaced with a fresh receiver solution.

### Metabolic Stability with Liver Microsomes from Mice/fFrom Humans

The metabolic degradation of the test compound was assayed at 37 °C with pooled liver microsomes from mice or from humans. The final incubation volume of 100 μL per time point contained 0.1 M potassium phosphate/2 mM MgCl_2_ pH 7.4 microsomal protein (0.5 mg/mL) and the test compound at a final concentration of 1 μM.

Following a short preincubation period at 37 °C, the reactions were initiated by the addition of beta-nicotinamide adenine dinucleotide phosphate, reduced form (NADPH, 1 mM), and terminated by transferring an aliquot into two equiv (v/v) of acetonitrile after each time point. Additionally, the NADPH-independent degradation was monitored in incubations without NADPH, which were terminated at the last time point. The [%] remaining test compound after NADPH-independent incubation is reflected by parameter c (control) (metabolic stability).

The quenched incubations were pelleted by centrifugation (10000*g*, 5 min). An aliquot of the supernatant was assayed by LC-MS/MS for the amount of the parent compound. The half-life (*t*_1/2_ *in vitro*) is determined by the slope of the semi-logarithmic plot of the concentration–time profile.

The intrinsic clearance, CL_int_ [μL min^–1^ (mg of protein)^−1^], was calculated by considering the amount of protein in the incubation:

$${\mathrm{CL}}_{\mathrm{int}}=\frac{\ln2}{t_{1/2}\cdot C_{\mathrm{protein}}}\cdot 1000$$

where *t*_1/2_ [min] is the half-life and *C*_protein_ [mg of protein/mL] is the protein content. CL_up,int_ [mL min^–1^ kg^–1^] is then calculated as

$${\mathrm{CL}}_{\mathrm{up},\mathrm{int}}=0.001\cdot {\mathrm{CL}}_{\mathrm{int}}\cdot W_{\mathrm{liver}}\cdot R\mathrm{mic}$$

where *W*_liver_ [g/kg b.w.] is the liver weight and *R*_mic_ [mg of protein/g of liver] is the recovery. The hepatic metabolic clearance, CL_hep,metab_ [mL min^–1^ kg^–1^], is calculated as follows:

$${\mathrm{CL}}_{\mathrm{hep},\mathrm{metab}}=\frac{Q\cdot {\mathrm{CL}}_{\mathrm{up},\mathrm{int}}}{Q+{\mathrm{CL}}_{\mathrm{up},\mathrm{int}}}$$

Results are expressed in terms of QH [%], the percentage of hepatic blood flow:

$$\mathrm{QH}=\frac{{\mathrm{CL}}_{\mathrm{hep},\mathrm{metab}}}{\mathrm{HBF}}\times 100\%$$

where HBF [mL min^–1^ kg^–1^] is the hepatic blood flow.

### Metabolic Stability in Human Hepatocytes

The metabolic degradation of test compounds was evaluated using cryopreserved human hepatocytes (BioIVT, 20-donor-pool, mixed gender) in suspension. Hepatocytes were recovered from cryopreservation and diluted in Dulbecco’s modified Eagle medium (supplemented with 7 μg/L glucagon, 5 mg/L insulin, 7.5 mg/L hydrocortisone (all from Merck), and 5% human serum) to obtain a final cell density of 1.0 × 10^6^ viable cells/mL. Following a preincubation in a cell culture incubator (37 °C, 10% CO_2_), test compounds dissolved in DMSO were added to the hepatocyte suspension, resulting in a final test compound concentration of 1 μM and a final DMSO concentration of 0.05%.

The cell suspension was incubated at 37 °C (cell culture incubator, horizontal shaker), and samples were removed from the incubation after 0, 0.5, 1, 2, 4, and 6 h. Samples were quenched with acetonitrile (containing an internal standard) and pelleted by centrifugation. The supernatant was transferred to a deep-well plate and prepared for HPLC-MS/MS analysis to monitor the test compound depletion.

The percentage of the remaining test compound was calculated based on the peak area ratio (test compound/internal standard) at each time point relative to the ratio at time 0. Log-transformed data were plotted against the incubation time, and the absolute value of the slope obtained via linear regression was used to estimate the *in vitro* half-life (*t*_1/2_).

The *in vitro* intrinsic clearance (CL_int,*in vitro*_) was calculated using the following equation:

$${\mathrm{CL}}_{\mathrm{int},\mathrm{in vitro}}=\frac{\ln2}{t_{1/2}\cdot H}$$

where *H* = 120 (10^6^ cells)/(g of liver) is the human hepatocellularity. CL_int,*in vitro*_ was scaled to the whole liver using human-specific parameters, including the liver factor (LF = 25.7 g of liver/kg of body weight) and HBF (21 mL min^–1^ kg^–1^).

The *in vivo* intrinsic clearance, CL_int,*in vivo*_ [mL min^–1^ kg^–1^], was derived using the following equation:

$${\mathrm{CL}}_{\mathrm{int},in\;vivo}=\frac{{\mathrm{CL}}_{\mathrm{int},in\;vivo}^{10^{6}\;\mathrm{cells}}\cdot H\cdot \mathrm{LF}}{1000}$$

where CL_int,*in vivo*_^10^6^ cells^ is the *in vivo* intrinsic clearance expressed in units of μL min^–1^ (10^6^ cells)^−1^.

The hepatic *in vivo* blood clearance, CL_hep,blood_ [mL min^–1^ kg^–1^], was predicted using the well-stirred liver model:

$${\mathrm{CL}}_{\mathrm{hep,blood}}=\frac{{\mathrm{CL}}_{\mathrm{int},in\;vivo}\cdot \mathrm{HBF}}{{\mathrm{CL}}_{\mathrm{int},in\;vivo}+\mathrm{HBF}}$$

### Measurement of Oxygen Consumption

Oxygen consumption rate (OCR) as an indicator of mitochondrial respiration was measured by using the XF Cell Mito Stress Test Kit in the Seahorse Extracellular Flux (XF96) Analyzer (Seahorse Bioscience Inc., North Billerica, MA). HEK293-Flp-in-hSLC13A5 cells and HEK293-Flp-in cells carrying an empty plasmid were seeded at 30,000 cells per well onto PS Cell Culture Microplates (Seahorse Biosciences) precoated with collagen type-1 in DMEM medium with 4.5 g/L glucose and 10% (v/v) fetal calf serum. Cells were maintained in the assay plate for 1 day before the measurement of cellular metabolism.

XF assay medium (XF Base Medium supplemented with 10 mM glucose and 2 mM sodium pyruvate) was freshly adjusted to pH 7.4 using sodium hydroxide. After respiration of the culture medium, all wells were washed three times with the assay medium. Following the addition of 180 μL of XF assay medium to each well, the plate was incubated in a 37 °C non-CO2 incubator for 1 h prior to measurement. The plate was then transferred to a Seahorse XF96 Analyzer for analysis. Once in XF96, HEK293-Flp-In-hSLC13A5 cells underwent measurements of basal oxygen consumption. Using XF96 4-port FluxPaks, successive treatments were performed with compound (variable concentrations with 0.1% DMSO)/DMSO solvent (0.1% final concentration), citrate (150 μM final concentration), and FCCP (carbonyl cyanide-ρ-trifluoromethoxyphenylhydrazone) (0.1 μM final concentration), each prediluted in XF assay medium.

All OCR measures were done four to six times in a 2–1–3 min mix–wait–measure cycle.

### Quantification and Statistical Analysis

#### Calculation of the Performance of Test Compounds in the 293-Flp-In Assay

The performance of the test compounds is calculated as follows: wells incubated with 1% DMSO only deliver cpm values for the noninhibited ^14^C-citrate uptake into the respective 293Flp-In cells (HIGH control; 100% CTL). Wells containing cells which have received 30 mM nonlabeled potassium citrate solution in addition to the ^14^C-citrate working solution deliver cpm values for the background (LOW control; 0% CTL). %CTL value per test compound concentration; %CTL (test compound) = (cpm value (test compound) – (cpm value (LOW control))/(cpm value (HIGH control) -– (cpm value (LOW control)). The IC50s of the test compounds were calculated in ExcelFit by sigmoidal dose–response analysis (variable slope). Data for both HIGH control (100% CTL, locked) and LOW control (0% CTL) are integrated as boundaries into the analysis. Inhibitors with activity on the uptake of ^14^C-citrate into the respective 293Flp-In cells are expected to deliver %CTL values significantly <100% CTL.

#### Calculation of the Performance of Test Compounds in the HepG2 Assay

The performance of the test compounds is calculated as follows: wells incubated with 1% DMSO only deliver cpm values for the noninhibited ^14^C-citrate uptake into HepG2 cells (HIGH control; 100% CTL). Wells that do not contain HepG2 cells but have received the common follow-up treatment (including the addition of ^14^C-citrate working solution) deliver cpm values for the background (LOW control; 0% CTL). %CTL value per test compound concentration; %CTL (test compound) = (cpm value (test compound) – (cpm value (LOW control))/(cpm value (HIGH control) – (cpm value (LOW control)). The IC50s of the test compounds were calculated in ExcelFit by sigmoidal dose–response (variable slope). Data for both HIGH control (100% CTL, locked) and LOW control (0% CTL) are integrated as boundaries into the analysis. NaCT inhibitors with activity on the uptake of ^14^C-citrate into HepG2 cells are expected to deliver %CTL values significantly <100% CTL. An optional lithium stimulation control is expected to deliver values clearly >100% CTL.

## Supplementary Material





## Notes

^1^ *g* NMR spectra for all final compounds, example HPLC profiles, and additional experimental data (PDF)
Molecular formula strings (CSV)

## ⊥

Diamond Light Source, Harwell Oxford, Didcot OX11 0DE, United Kingdom

## #

Pharmaceutical & Biological Chemistry, UCL, Gower Street, London, UK, WC1E 6BT

## ∇

Action Against Age-related Macular Degeneration, 3 Queen Square, London—UK, WC1N 3AR

## ○

Weatherall Institute of Molecular Medicine, Radcliffe Department of Medicine, University of Oxford, UK, OX3 9DU

## Abbreviations

ADME: absorption, distribution, metabolism, and excretion
CNS: central nervous system
cpm: counts per minute
DSF: differential scanning fluorimetry
NMR: nuclear magnetic resonance
PAMPA: passive permeability in the parallel artificial membrane permeability assay
PE: permeability coefficients
SEC: size exclusion chromatography
SLC13A5: NaCT, sodium-citrate transporter
