# Supplementary material for: Discovery and Characterization of a Nonacidic Small-Molecule Inhibitor of the Sodium-Coupled Dicarboxylate Transporter NaCT
Source: J Med Chem. 2026 Apr 28;69(9):10046–59. doi: 10.1021/acs.jmedchem.5c02711 (PMC13181786; doi:10.1021/acs.jmedchem.5c02711)
Supplement: Supplementary file 1 [file jm5c02711_si_001.pdf]

Supporting Information

Discovery and characterization of a non-acidic small-molecule inhibitor of the sodium coupled di-carboxylate transporter NaCT.

Andrew Quigley[†‡]*, Jörg T. Kley[§]*, Alexander Pautsch[§], Stefan G. Kauschke[‖], Amy Chu-Antypas [†], Annamaria Tessitore[†], Claire Strain-Damerell[†⊥], Leela Shrestha[†ø], Dieter Wiedenmayer[§], Nicola A. Burgess-Brown[†Ψ], Oleg Fedorov[†], Elisabeth P. Carpenter[Φ†]*

AUTHOR ADDRESS

[†]Centre for Medicines Discovery, NDM Research Building, Roosevelt Dr, Headington, Oxford OX3 7FZ.

[‡]Membrane Protein Laboratory, Research Complex at Harwell, Diamond Light Source, Harwell Oxford, Didcot OX11 0DE, United Kingdom

[§]Global Medicinal Chemistry, Boehringer Ingelheim Pharma GmbH & Co. KG, 88397 Biberach, Germany

[‖]Cardio-Renal-Metabolic Disease Discovery Research, Boehringer Ingelheim Pharma GmbH & Co. KG, 88397 Biberach, Germany

Corresponding Authors

*****_andrew.quigley @diamond.ac.uk; joerg.kley@ boehringer-ingelheim.com; lizcarpen1@gmail.com_

Classification: Confidential - General

Table of Contents

Table S1: Structures and citrate uptake inhibition data of compounds (1), (2), (4) – (8) and selected further piperidinecarboxamides. s3

Table S2 Cerep® SafetyScreen44™ data for BI01383298 (6) (single point measurements @10 µM). Based on cellular potency of 50 nM (HEK293-Flp-in-hNaCT) and 24.5 nM (HepG2), % control values @10 µM of roughly >40% indicate >100-fold selectivity. s5

Table S3 Analysis of vcINDY thermostability in the presence and absence of potential inhibitors and substrates. s7

Figure S1: Representative unfolding response of hNaCT alone and in the presence of lithium citrate, sodium citrate, 1% DMSO, BI01383298 (6) and BI01372674 (7). Upper panel show integrated fluorescence with the lower panel showing the first derivative of the integrated fluorescence. s8

Figure S2. Structural model of NaCT (PDB: 7JSK) highlighting the citrate-binding pocket and the predicted interaction of BI01383298 (6). Citrate is displayed in dark blue with its hydrogen-bonding interactions shown in cyan. The five highest-ranked ligand poses predicted by Boltz2 are overlaid and depicted in complementary pastel colors. Sodium ions present in the 7JSK structure are shown in purple. s9

Figure S3: ^{1}H NMR of BI01383298 (6) s10

Figure S4: ^{13}C NMR of BI01383298 (6) s10

Figure S5: HPLC trace of compound (6) s11

Figure S6: ^{1}H NMR of BI01372674 (7). s12

Figure S7: ^{13}C NMR of BI01372674 (7). s12

Figure S8: HPLC trace of BI01372674 (7). s13

Figure S9: ^{1}H NMR of compound (8). s14

Figure S10: ^{13}C NMR of compound (8). s14

Classification: Confidential - General

Table S1: Structures and citrate uptake inhibition data of compounds (1), (2), (4) – (8) and selected further piperidinecarboxamides.

Compound and code	Structure	HepG2 IC_{50} [μM]
PF-06649298 **(1)**	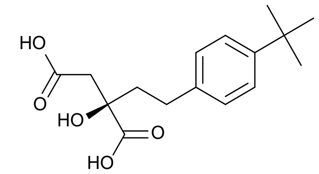	13.3
Salicylanilide **(2)**	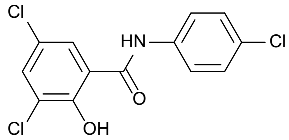	0.028
Piperidine-carboxamide **(4)**	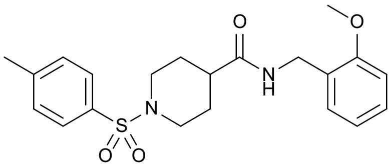	5.03
Piperidine-carboxamide **(5)**	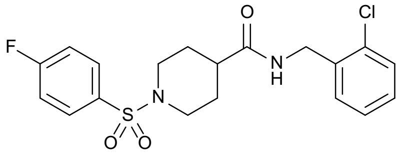	4.62
BI01383298 **(6)**	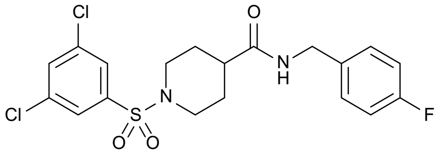	0.025
BI01372674 **(7)**	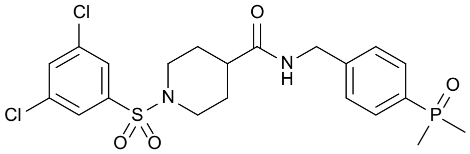	>100
BI01455810 **(8)**	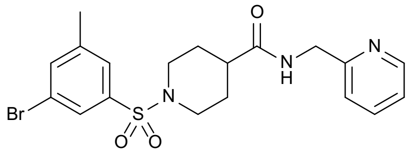	0.184
**(9)**	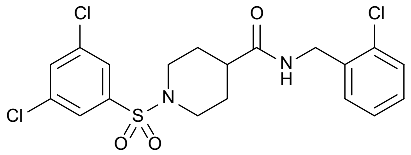	0.060
**(10)**	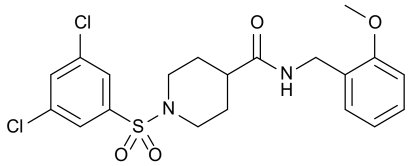	0.065
**(11)**	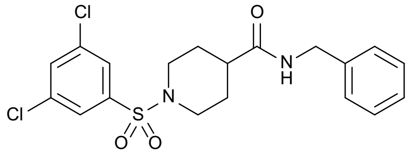	0.040

Classification: Confidential - General

**(12)**	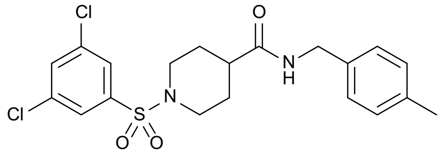	0.037
**(13)**	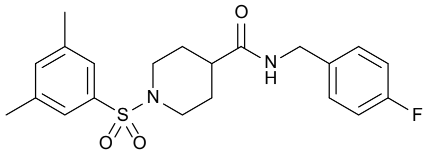	0.035
**(14)**	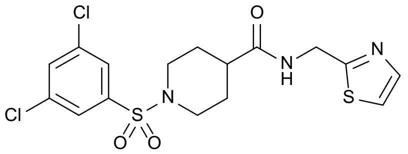	0.246
**(15)**	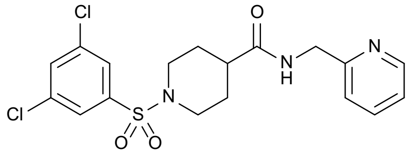	0.455
**(16)**	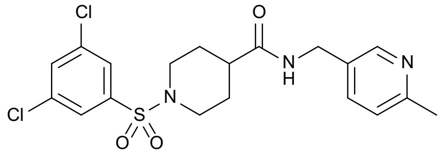	0.632[a]
**(17)**	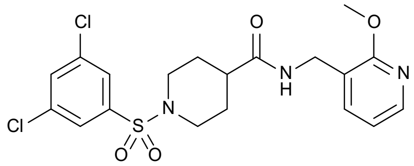	0.201[a]
**(18)**	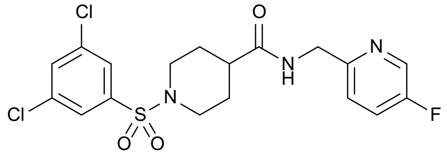	0.233

[a]n=1 (all other values are based on at least two independent measurements)

Classification: Confidential - General

Table S2 Cerep® SafetyScreen44™ data for BI01383298 (6) (single point measurements @10 µM). Based on cellular potency of 50 nM (HEK293-Flp-in-hNaCT) and 24.5 nM (HepG2), % control values @10 µM of roughly >40% indicate >100-fold selectivity.

HGNC ID	SafetyScreen44™ Screen Name	Species	% control _(10uM)	SafetyScreen44™ Target type
HTR1A	5HT1A/H	*H. sapiens*	105	G-Protein Coupled Receptor Class A
HTR1B	5HT1B/R	*R. norvegicus*	99	G-Protein Coupled Receptor Class A
HTR2A	5HT2AH_AGON	*H. sapiens*	85	G-Protein Coupled Receptor Class A
HTR2B	5HT2B/H AG	*H. sapiens*	90	G-Protein Coupled Receptor
HTR3A	5HT3/H	*H. sapiens*	103	Ligand-gated ion channels
ADORA2A	A2A/H	*H. sapiens*	111	G-Protein Coupled Receptor Class A
ACE	ACE(HU_AMTCH400)	*H. sapiens*	103	
ADRA1A	ALPHA1AH_ANTAG	*H. sapiens*	95	G-Protein Coupled Receptor Class A
ADRA2A	ALPHA2A/HU	*H. sapiens*	91	G-Protein Coupled Receptor Class A
AR	ANDROGEN/H	*H. sapiens*	86	Steroid hormone receptors
ADRB1	BETA1/HUM	*H. sapiens*	110	G-Protein Coupled Receptor Class A
ADRB2	BETA2/HUM	*H. sapiens*	96	G-Protein Coupled Receptor Class A
GABRA1	BZD/CENTR/R	*R. norvegicus*	113	Ligand-gated ion channels
CACNA1C	CA+/DHPSI/R	*R. norvegicus*	93	Voltage-gated ion channels
CNR2	CB2/PERIPH/H	*H. sapiens*	106	G-Protein Coupled Receptor Class A
CCKAR	CCKA/H	*H. sapiens*	77	G-Protein Coupled Receptor Class A
CNR1	CNR1	*H. sapiens*	22	G-Protein Coupled Receptor Class A
PTGS1	COX-1	*H. sapiens*	95	Hydrolase under EC 3
PTGS2	COX-2	*H. sapiens*	119	Hydrolase under EC 3
DRD1	D1/H	*H. sapiens*	94	G-Protein Coupled Receptor Class A
DRD2	D2SH_AGON	*H. sapiens*	90	G-Protein Coupled Receptor Class A
SLC6A3	DATRANS/HUM	*H. sapiens*	91	SLC superfamily of solute carriers
OPRD1	DELTA2/H	*H. sapiens*	99	G-Protein Coupled Receptor Class A
EDNRA	ETA/H	*H. sapiens*	94	G-Protein Coupled Receptor Class A

Classification: Confidential - General

NR3C1	GCORTICOID/H	*H. sapiens*	98	Steroid hormone receptors
HRH1	H1/PYRIL/HS	*H. sapiens*	107	G-Protein Coupled Receptor Class A
HRH2	H2/APT/HS	*H. sapiens*	106	G-Protein Coupled Receptor Class A
KCNH2	HERG_DOFETILIDE	*H. sapiens*	83	Voltage-gated ion channels
KCNA1	K+/VOLT/RA	*R. norvegicus*	97	Voltage-gated ion channels
LCK	LCK_CE	*H. sapiens*	103	Kinase under EC2.7
CHRM1	M1/H	*H. sapiens*	98	G-Protein Coupled Receptor Class A
CHRM2	M2/H	*H. sapiens*	98	G-Protein Coupled Receptor Class A
CHRM3	M3/H	*H. sapiens*	100	G-Protein Coupled Receptor Class A
MAOA	MAO-A_ANTAG	*R. norvegicus*	85	Oxidoreductase under EC 1
OPRM1	MU/H	*H. sapiens*	74	G-Protein Coupled Receptor Class A
CHRNA4	N_NEURO_A4B2	*H. sapiens*	113	G-Protein Coupled Receptor Class A
SCN4A	NA+/SITE2/R	*R. norvegicus*	48	Voltage-gated ion channels
SLC6A2	NEUP/H	*H. sapiens*	93	SLC superfamily of solute carriers
GRIN1	NMDA/R	*R. norvegicus*	102	Ligand-gated ion channels
OPRK1	OPRK1	*R. norvegicus*	19	G-Protein Coupled Receptor Class A
PDE3A	PDE3A	*H. sapiens*	80	Hydrolase under EC 3
PDE4D	PDE4D2	*H. sapiens*	105	Hydrolase under EC 3
SLC6A4	SLC6A4/H	*H. sapiens*	92	SLC superfamily of solute carriers
AVPR1A	V1A/HUM	*H. sapiens*	89	G-Protein Coupled Receptor Class A

Classification: Confidential - General

Table S3 Analysis of vcINDY thermostability in the presence and absence of potential inhibitors and substrates.

Sample	T_m measurement 1	T_m measurement 2	Mean T_m (n=2)	Δ Tm
Apo/DMSO	52.5°C	52.7°C	52.6°C	-
Apo	52.4°C	52.4°C	52.4°C	-0.2
PF-06649298 **(1)**	53.5°C	53.0°C	53.3°C	0.7
BI01383298 **(6)**	52.5°C	52.3°C	52.4°C	-0.2
BI01372674 **(7)**	52.4°C	52.5°C	52.5°C	-0.1
BI01455810 **(8)**	50.6°C	52.3°C	51.5°C	-1.1
Sodium succinate	73.1°C	72.4°C	72.8°C	20.2
Lithium citrate	61.7°C	61.2°C	61.5°C	8.9

Classification: Confidential - General

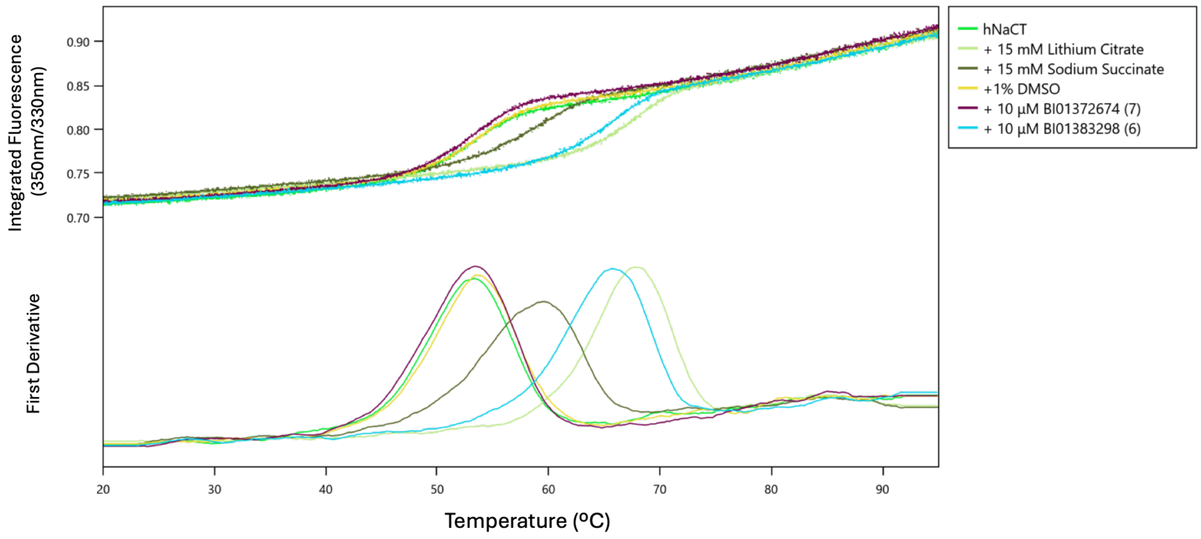

Figure S1: Representative unfolding response of hNaCT alone and in the presence of lithium citrate, sodium citrate, 1% DMSO, BI01383298 (6) and BI01372674 (7). Upper panel show integrated fluorescence with the lower panel showing the first derivative of the integrated fluorescence.

Classification: Confidential - General

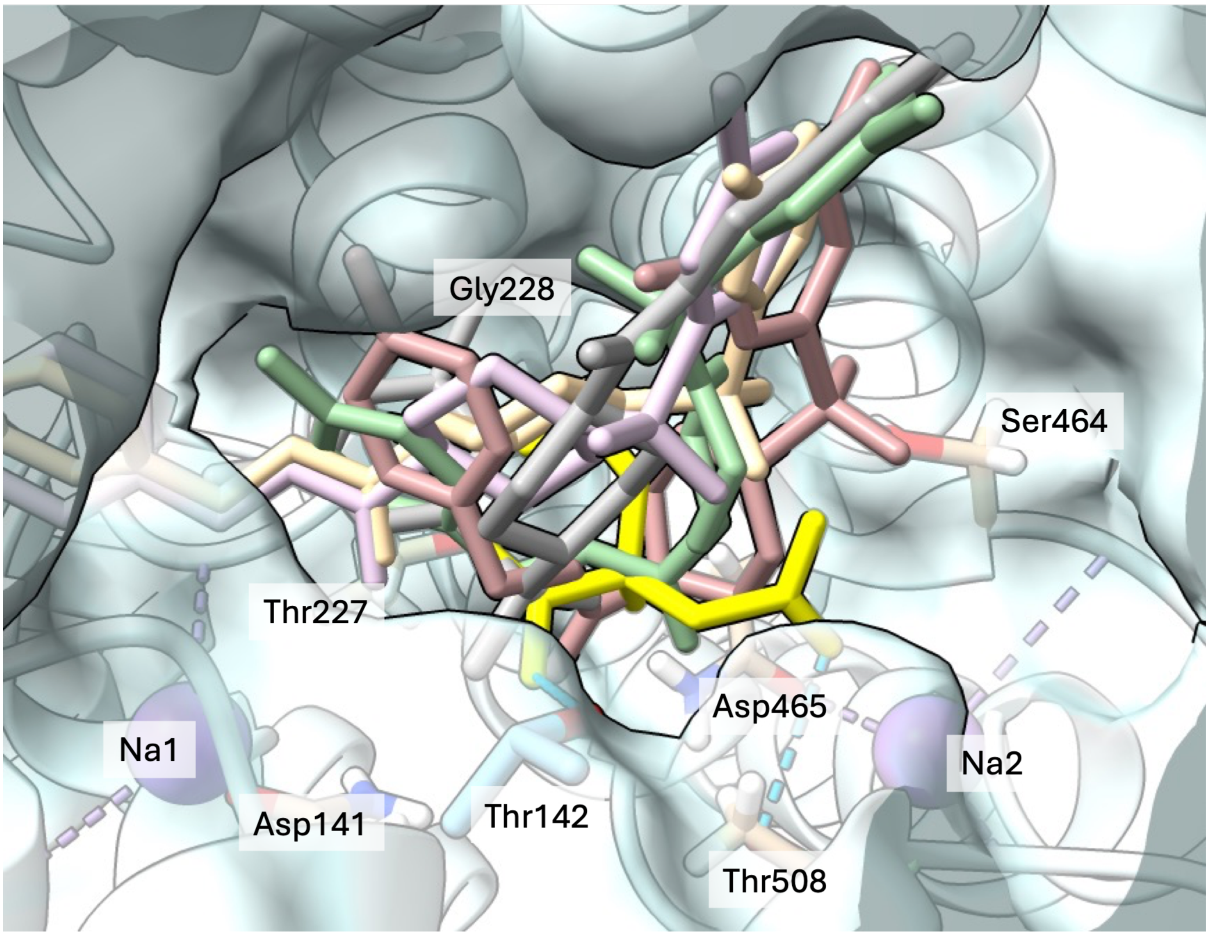

Figure S2. Structural model of NaCT (PDB: 7JSK) highlighting the citrate-binding pocket and the predicted interaction of BI01383298 (6). Citrate is displayed in yellow. Labelled residues form the citrate binding site. The five highest-ranked ligand poses predicted by Boltz2 (version 2.2.0) are overlaid and depicted in complementary pastel colors. Sodium ions are shown in purple. pLDDT scores for models where between 0.858 and 0.868. PTM scores were between 0.881 and 0.893. iPTM score between 0.859 and 0.882. The combined confidence score was between 0.858 and 0.870. The protein sequence was taken from Unitprot ID Q86YT5-1.

Classification: Confidential - General

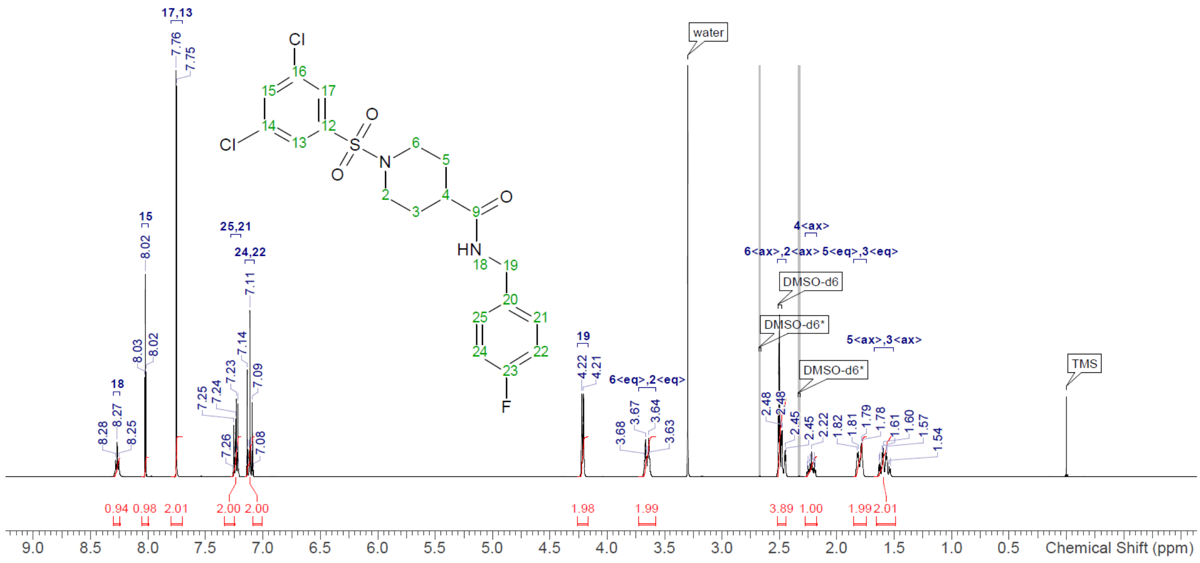

Figure S3: ^{1}H NMR of BI01383298 (6)

^{13}C NMR (101 MHz, *DMSO-d_6*) δ ppm 173.3 (s) 161.0 (d, *J*=242.2 Hz) 139.2 (s) 135.7 (d, *J*=2.6 Hz) 135.3 (s) 132.7 (s) 128.9 (d, *J*=8.2 Hz) 125.7 (s) 114.9 (d, *J*=21.1 Hz) 45.2 (s) 41.2 (s) 27.6 (s)

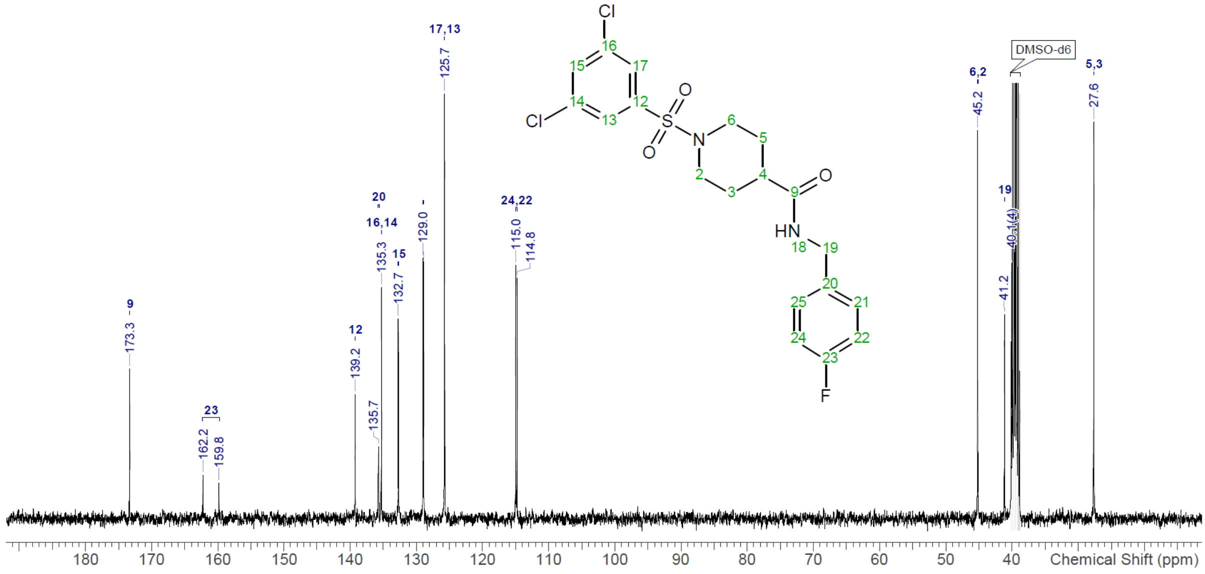

Figure S4: ^{13}C NMR of BI01383298 (6)

Classification: Confidential - General

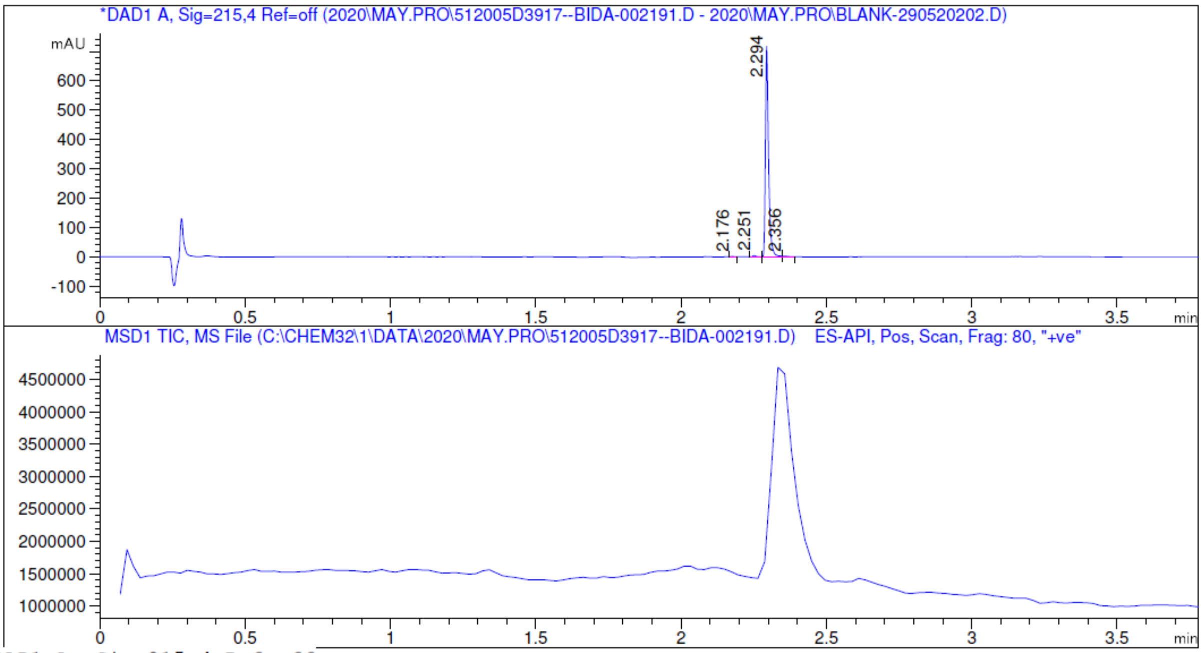

DAD1 A, Sig=215,4 Ref=off

PEAK No	RT min	Height	Area	Area %
1	2.176	1.086	0.728	0.132
2	2.251	4.627	3.353	0.608
3	2.294	719.260	544.818	98.827
4	2.356	2.215	2.383	0.432

Figure S5: HPLC trace of compound (6)

Classification: Confidential - General

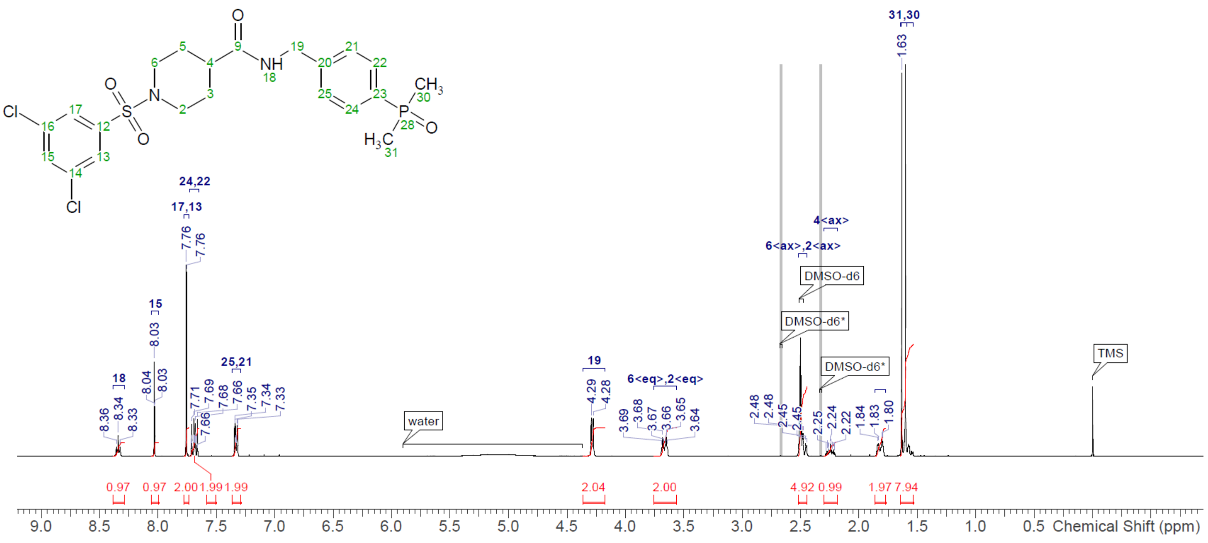

Figure S6: ^{1}H NMR of BI01372674 (7).

^{13}C NMR (101 MHz, *DMSO-d*$_6$) δ ppm 173.4 (s) 143.0 (d, *J*=2.6 Hz) 139.2 (s) 135.3 (s) 134.3 (br d, *J*=96.4 Hz) 132.7 (s) 129.7 (d, *J*=10.3 Hz) 127.0 (d, *J*=11.6 Hz) 125.8 (s) 45.2 (s) 41.7 (s) 40.1 (s) 27.7 (s) 17.7 (d, *J*=70.6 Hz)

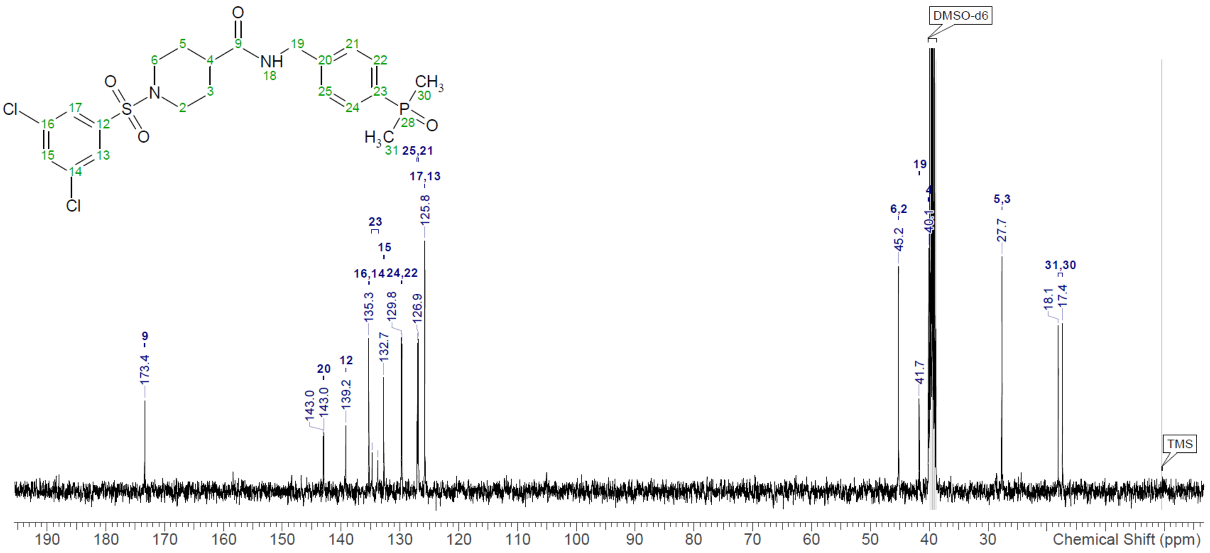

Figure S7: ^{13}C NMR of BI01372674 (7).

Classification: Confidential - General

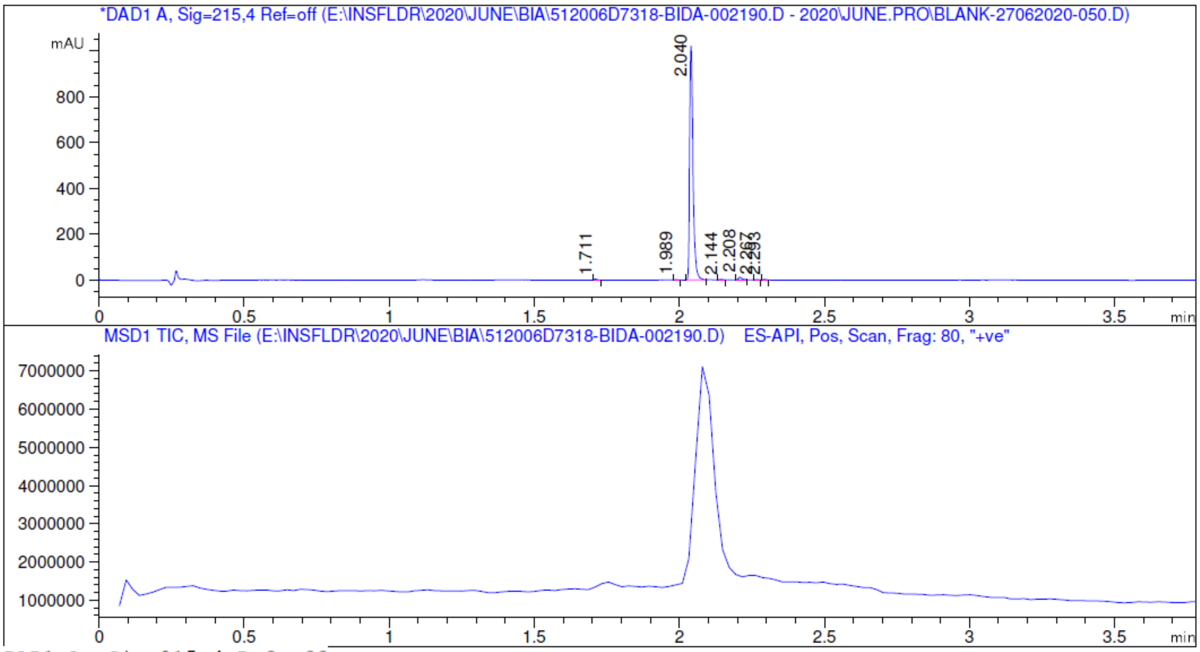

DAD1 A, Sig=215,4 Ref=off

PEAK No	RT min	Height	Area	Area %
1	1.711	4.770	3.640	0.431
2	1.989	1.288	0.921	0.109
3	2.040	1.024e3	825.502	97.692
4	2.144	2.515	2.146	0.254
5	2.208	11.453	9.752	1.154
6	2.267	1.519	1.223	0.145
7	2.293	2.570	1.817	0.215

Figure S8: HPLC trace of BI01372674 (7).

Classification: Confidential - General

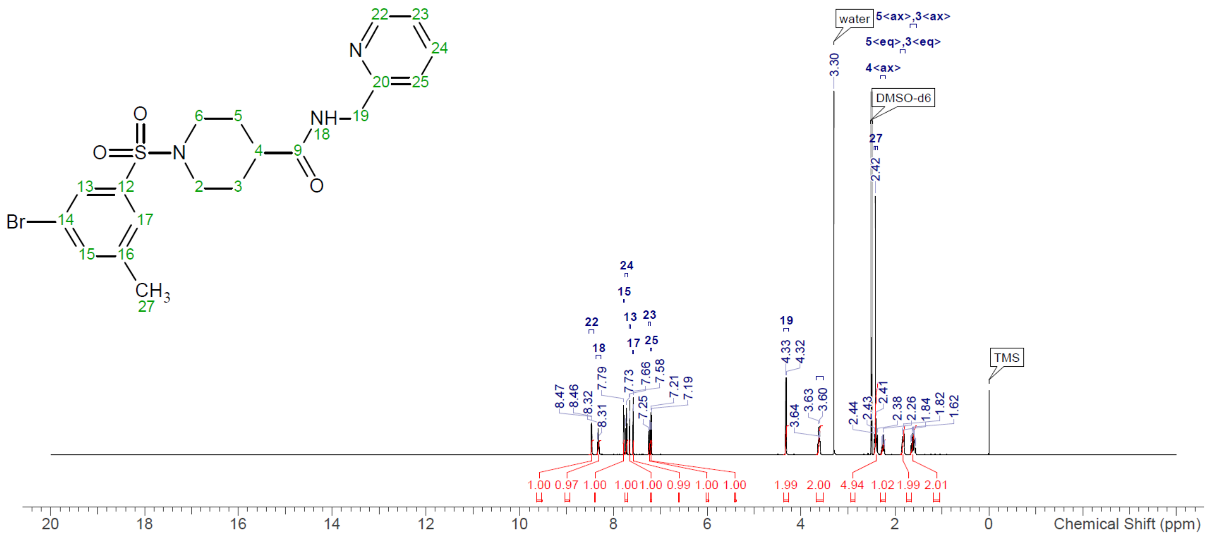

Figure S9: ^{1}H NMR of compound (8).

^{13}C NMR (101 MHz, *DMSO-d$_6$*) δ ppm 173.5 (s) 158.6 (s) 148.7 (s) 142.1 (s) 137.5 (s) 136.6 (s) 136.2 (s) 126.7 (s) 126.6 (s) 122.0 (s) 121.9 (s) 120.7 (s) 45.3 (s) 44.0 (s) 40.1 (s) 27.7 (s) 20.4 (s)

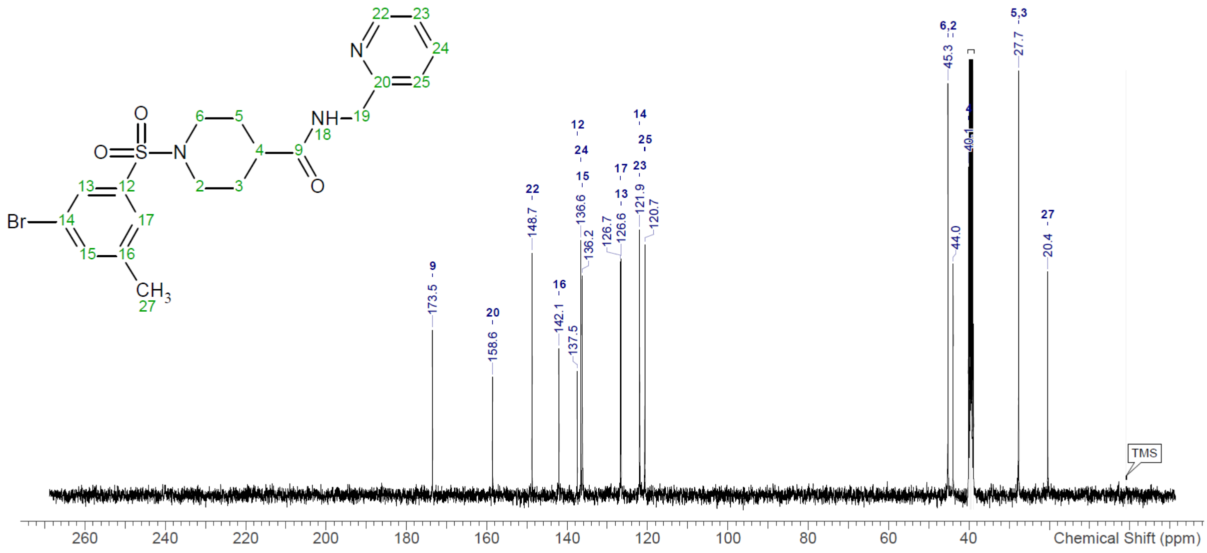

Figure S10: ^{13}C NMR of compound (8).

Classification: Confidential - General
